# AR antagonists develop drug resistance through TOMM20 autophagic degradation-promoted transformation to neuroendocrine prostate cancer

**DOI:** 10.1186/s13046-023-02776-0

**Published:** 2023-08-10

**Authors:** Linglong Yin, Yubing Ye, Ling Zou, Jinli Lin, Yi Dai, Yongming Fu, Youhong Liu, Yuchong Peng, Yingxue Gao, Yuxin Fu, Xuli Qi, Tanggang Deng, Songwei Zhang, Xiong Li

**Affiliations:** 1 Key Laboratory of Clinical Precision Pharmacy of Guangdong Higher Education Institutes, The First Affiliated Hospital, Guangdong Pharmaceutical University, 19 Nonglinxia Road, Yuexiu District, Guangzhou, Guangdong China; 2 Clinical Pharmacy, The First Affiliated Hospital, Guangdong Pharmaceutical University, Guangdong, China; 3 School of Clinical Pharmacy, Guangdong Pharmaceutical University, Guangdong, China; 4 Department of Oncology, Center for Molecular Medicine, Xiangya Hospital, Central South University, Changsha, China; 5 Hunan Key Laboratory of Molecular Radiation Oncology, Xiangya Hospital, Central South University, Changsha, China; 6 NMPA Key Laboratory for Technology Research and Evaluation of Pharmacovigilance, Guangdong Pharmaceutical University, Guangdong, China

**Keywords:** AR Antagonists, Drug Resistance, TOMM20, Autophagic Degradation, Neuroendocrine Prostate Cancer

## Abstract

**Background:**

Prostate cancer(PCa) is the most commonly occurring male cancer in the USA. Abiraterone or Enzalutamide have been approved for the treatment of metastatic castration-resistant prostate cancer (CRPC). However, the treatment-emergent neuroendocrine PCa (t-NEPC) may develop, resulting in drug resistance in about 10–17% CRPC patients. The detailed mechanisms remain unclear..

**Methods:**

The expression correlation of TOMM20 and AR in PCa was determined by analyzing publicly available datasets, or by IHC staining in tumor specimens. The protein interaction of TOMM20 and AR was validated by co-immunoprecipitation or GST pull-down assay. The impact of TOMM20 depletion on drug sensitivity were elucidated by assays of cell proliferation, invasion, sphere formation, xenograft growth and intravenous metastasis. The intracellular ROS level was measured by flow cytometry, and the NEPC transdifferentiation and characteristics of cancer stem-like cells were validated by RNA-seq, RT-PCR and western blotting.

**Results:**

The protein level of TOMM20 is positively correlated with AR in PCa cells and specimens. TOMM20 protein physically interacts with AR. AR antagonists induced the protein degradation of TOMM20 through autophagy-lysosomal pathway, thereby elevating the intracellular ROS level and activating PI3K/AKT signaling pathway. When TOMM20 was depleted, PCa cells underwent EMT, acquired the characteristics of cancer stem-like cells, and developed resistance to AR antagonists. The stable depletion of TOMM20 promoted the transdifferentiation of PCa adenocarcinoma into NEPC and metastasis. Conversely, the rescue of TOMM20 re-sensitized the resistant PCa cells to AR antagonists.

**Conclusions:**

TOMM20 protein degradation induced by AR antagonists promoted the transdifferentiation of PCa to NEPC, thereby revealing a novel molecular mechanism by which AR antagonists develop drug resistance through mitochondrial outer membrane-mediated signaling pathway. These findings suggested that the decreasing or loss of TOMM20 expression in PCa tissues might become a useful predictor of PCa resistance to AR antagonists.

**Supplementary Information:**

The online version contains supplementary material available at 10.1186/s13046-023-02776-0.

## Background

Prostate cancer (PCa) is the male cancer type with the highest incidence and the second leading cause of mortality in the western counties [1]. In China, PCa has become the leading urogenital tumor and the 6^th^ most prevalent male tumors. There were 115,426 new PCa patients and 51,094 PCa deaths in China in 2020 [2].

Androgen/AR plays a critical role in the initiation and progression of PCa, and the protein stability of AR is required to regulate gene transcription. Before binding to androgen, cytoplasmic AR maintains protein stability by forming complexes with molecular chaperones like HSP70, HSP90, HSP40 or HSP27 [3–7]. When activated by androgen, the phosphorylated AR translocates to the nucleus, binds to the AR-response elements (ARE) in the promoter/enhancer of target genes, and regulates gene transcription [8]. Therefore, androgen deprivation therapy (ADT) becomes the first-line modality in PCa and several AR antagonists have been widely used in clinic to inhibit androgen-mediated activation of AR. However, PCa patients inevitably develop drug resistance within 12–18 months, and eventually progress to the stage of metastatic castration-resistant PCa (mCRPC) [9]. It has been reported that ADT resistance develops due to AR gene amplification, AR genomic mutations, and the generation of AR splicing such as ARV7 or ARV9 [10, 11]. Additionally, alternative mechanisms independent of AR, such as abnormal hormonal secretion in the tumor microenvironment, DNA damage repair (DDR), phosphoinositide 3-kinase–protein kinase B–mammalian target of rapamycin (PI3K–AKT–mTOR), and Wnt–β-catenin, play critical roles in the development of ADT resistance [12–14].

Second-generation AR antagonist Enzalutamide and CYP17A1 inhibitor Abiraterone have been approved for the treatment of metastatic PCa [15, 16], but their efficacy is limited by the acquired drug resistance. Recent studies have implicated that the transdifferentiation of adenocarcinoma into treatment-emergent neuroendocrine PCa (t-NEPC) is a critical mechanism of drug resistance after treatment with the second generation AR antagonists [17–21]. It is estimated that approximately 10–17% of mCRPC patients develop t-NEPC after AR antagonist treatment, and the clinical outcome is quite poor [22]. In the tumor tissues of NEPC patients, AR signaling is low or lost, and such typical NE markers as neuronal-specific enolase 2 (ENO2), synaptophysin (SYP), NCAM1 and chromogranin A(CHGA) are remarkably elevated [23]. The molecular mechanisms underlying the progression of NEPC include the loss of tumor suppressor genes p53 or Rb, abnormal epigenetic modifications, and the hyperactivation of such oncogenic transcription factors as SOX2 and BRN2 [24, 25]. In the transition to t-NEPC, PCa tumors exhibit the reactivation of developmental programs associated with epithelial–mesenchymal plasticity and acquisition of cancer stem-like cell properties [26].

Recent reports indicated that AR protein is located in the mitochondria of PCa cells [27–29], and ectopic AR expression reduced the expression of mitochondrial oxidative phosphorylation complex subunits. Translocase of the outer mitochondrial membrane complex subunit 20 (TOMM20) is a structural protein of mitochondria responsible for the recognition and translocation of mitochondrial proteins inside the mitochondria from the cytosol [30]. TOMM20 is highly correlated with the degree of malignancy of several cancer types, including PCa [27, 31–36]. However, the roles of TOMM20 in PCa progression remain unknown, particularly in the transformation to NEPC. In the present study, we first found a correlation between TOMM20 and AR protein levels, and that TOMM20 protein physically interacts with AR. AR antagonists induced the autophagic protein degradation of TOMM20, thereby elevating the ROS-activated PI3K/AKT signaling pathway. When TOMM20 was depleted, the PCa cells underwent EMT, acquired the characteristics of cancer stem-like cells, and developed resistance to AR antagonists. The stable depletion of TOMM20 promoted the transdifferentiation of PCa adenocarcinoma cells into NEPC. The present study identified the novel roles of TOMM20 protein degradation in NEPC transdifferentiation, and elucidated a novel molecular mechanism by which AR antagonists develop acquired drug resistance.

## Materials and methods

### Cell lines and cell culture

Human PCa cells LNCaP, VCaP, PC-3, DU145, NEPC cells NCI-H660,HEK293T cells and non-malignant prostate epithelial cells RWPE-1 were purchased from ATCC (Manassas, VA, USA). C4–2 and CWR22Rv1 were obtained from Dr. Chinghai Kao at the Indiana University School of Medicine. PCa cells were maintained in RPMI-1640 medium (Thermo Fisher Scientific, Waltham, MA, USA) supplemented with 10% FBS (Gibco, ThermoFisher Scientific, Friendship, ME, USA) and penicillin/streptomycin antibiotics. HEK293T cells were cultured in DMEM (Thermo Fisher Scientific, Waltham, MA, USA,with/without Sodium Pyruvate) medium and supplemented with 10% FBS (Gibco, ThermoFisher Scientific, Friendship, ME, USA) and penicillin/streptomycin antibiotics.RWPE-1 cells were maintained in defined Keratinocyte-SFM (1 ×) liquid (Invitrogen, Carlsbad, CA, USA). NCI-H660 cells were maintained according to the manufacturers’protocols.All cells were cultured with 5% CO2 in a 37 °C incubator.

### Lentivirus packaging and infection

The lentivirus system was applied to introduce the shRNAs or cDNAs into PCa cells. HEK-293 T cells were co-transfected with the virus package plasmids psAX2, pMD2G, and the gene expression plasmids. After 48 h transfection, the supernatants consisting of viral particles were collected for immediate use and/or frozen at -80℃ for later use.

### Transient transfection

pcDNA3.1-His/Myc-TOMM20 and pCMV-AR-Flag plasmids were constructed as follows. The cDNA fragments of TOMM20 and AR were generated individually using PCR and then subcloned into the described vectors. The constructed DNA fragments were confirmed by sequencing. Plasmids pcDNA3.1-His/Myc-TOMM20(∆TPR) and pcDNA3.1-His/Myc-TOMM20(∆Glu) were purchased from Genscript ProBio (Nanjing,China).

siRNA transfection was performed using the Mirus transfection kit according to the manufacturer’s instructions (Mirus, Madison, WI, USA). Non-targeting (NT) siRNA was used as the control. The sequences of siRNA (5′-3′) are listed in the Supplementary Table 1.

### Real time quantitative PCR

Total RNA was extracted using TRIzol (Takara Bio Inc.) and 1 µg of total RNA was used for cDNA synthesis using the Super Script III First Strand Synthesis System (Invitrogen) according to the manufacturer’s instructions. Quantitative real-time PCR (qRT-PCR) was conducted using a Bio-Rad CFX96 system with SYBR green to detect the mRNA expression level of a gene of interest. Expression levels were normalized to GAPDH. The primers used for the genes of interest are listed in Supplementary Table 1.

### Chemicals and reagents

Cycloheximide (CHX) and Bafilomycin A1 were purchased from Cell Signaling Technology (CST). Hydroxychloroquine Sulfate(HCQ), Enzalutamide (MDV3100) and Bicalutamide(Casodex) were purchased from Selleck, China (Shanghai, China). MK2206 was purchased from MedChemExpress (MCE, Shanghai, China). MG132 was purchased from Targetmol,USA (T2154).All were dissolved in dimethylsulfoxide (DMSO) except for HCQ dissolved in water.

### Antibodies

AR(rabbit monoclonal, 1:1000, #5153), PSA/KLK3(rabbit monoclonal, 1:1000, #5365), TOMM20(rabbit monoclonal, 1:1000, #42,406), ATG5(rabbit monoclonal, 1:1000, #12994S), LC3I/II(rabbit monoclonal, 1:1000, #12,741), Akt (rabbit monoclonal, 1:1000, #4691), Phospho-Akt (Ser473)(rabbit monoclonal, 1:1000, #4060), Phospho-Akt (Thr308)(rabbit monoclonal, 1:1000, #13,038), Nanog(rabbit monoclonal, 1:1000, #4903), Sox2(rabbit monoclonal, 1:1000, #3579), ALDH1A1(rabbit monoclonal, 1:1000, #36671S) and VDAC(rabbit monoclonal, 1:1000, #4661) antibodies were purchased from Cell Signaling Technology (CST). NCAM1(rabbit polyclonal antibody, 1:1000, A7913), β-Actin(mouse polyclonal, 1:5000, AC004) and α-Tublin(mouse polyclonal, 1:5000, AC012) antibodies were purchased from ABclonal Technology (Upper Heyford, UK). NSE (rabbit monoclonal, 1:1000, ab180943) and Syp (Rabbit monoclonal, 1:1000, ab184176) were purchased from Abcam (Shanghai, China). ALDH1A1(mouse monoclonal antibody, 1:500,sc-166362), TOMM70(mouse monoclonal antibody, 1:500, sc-390545),AR(rabbit polyclonal, 1:1000, Sc-815X) antibodies were purchased from Santa cruz. E-cadherin (rabbit polyclonal, 1:1000, GTX100443),BRN2(rabbit polyclonal, 1:1000, GTX114650) and N-cadherin (rabbit polyclonal, 1:1000, GTX127345) were purchased from Genetex.

### Cell immunofluorescence

LNCaP and C4-2 cells were maintained in 24-well plates (SORFA, Huzhou, China) with coverslips for 48 h. Cells were fixed with 4% paraformaldehyde for 10 min and permeabilized with 0.1% Triton X-100 for addtional 10 min. Cells were then incubated with AR and TOMM20 antibodies at 4 °C overnight. Cells were washed with cold PBS for three times, and then incubated with the second antibody. The nuclei were stained with DAPI. Images were captured and analyzed with a confocal laser scanning microscope.

### Western blot analysis and immunoprecipitation

Cells were lysed in lysis buffer and proteins (10–30 µg) were separated on 8–10% SDS/PAGE gel and then transferred onto PVDF membranes (Millipore, Billerica, MA). After blocking membranes, they were incubated with primary antibodies, HRP-conjugated secondary antibodies, and visualized using the ECL system (Thermo Fisher Scientific, Rochester, NY).

Extracts for immunoprecipitation were prepared using RIPA buffer supplemented with protease inhibitor cocktails. After centrifugation, 100 µl of the supernatants were prepared as input. The extracts were incubated with the indicated antibodies at 4℃ overnight on a rotator, followed by incubation with protein A/G-magnetic beads (MCE) at 4℃ for 2 h on a rotator. After incubation, beads were washed three times in immunoprecipitation buffer and boiled in 1 × loading buffer. Protein samples were analyzed by SDS-PAGE.

### GST pull-down assay

pGEX-4 T-1-AR(FL), pGEX-4 T-1AR(∆NTD), pGEX-4 T-1-AR (DBD) and pGEX-4 T-1-AR(∆LBD) were constructed using the GST gene fusion system according to the manufacturer’s instructions. To produce glutathione S-transferase(GST), GST-AR(FL), GST-AR(∆NTD), GST-AR(∆DBD) and GST-AR (∆LBD)fusion proteins, pGEX-4 T-1, pGEX-4 T-1-AR(FL), pGEX-4 T-1AR(∆NTD), pGEX-4 T-1-AR(∆DBD) or pGEX-4 T-1-AR(∆LBD) was individually transfected into BL21 competent cells. Protein expression was induced by ispropylb-D-1-thiogalactopyranoside (IPTG). GST-AR(FL), GST-AR(∆NTD), GST-AR(∆DBD) and GST-AR(∆LBD) and GST proteins were purified by binding to glutathione-sepharose resins (Sangon Biotech, Shanghai, China). Resins were incubated with cell lysates from LNCaP cells at 4 °C for 12 h on a rotator, and then were washed with the washing buffer four times. Resin-bound complexes were eluted by boiling, and subjected to western blotting.

### Clonogenic formation assay

The cells were plated in 6-well plates (500 cells/well) at 37℃ overnight and then treated with Abiraterone or Casodex. Cell culture media were replaced with fresh growth medium containing drugs every 3 days. The cells were fixed and stained with 0.1% crystal violet solution 7 days after drug treatment. Clones with > 50 cells were counted under the microscope and the survival fractions were calculated as the average number of colonies ± SD of three independent experiments.

### Cell viability assay and invasion assay

Cell viability was assessed by MTT assay. Cells were plated in 96-well plates with a cell density of 5 × 10^3^ cells per well, and incubated at 37℃ for 0, 24,48, 72, and 96 h. The culture medium was replaced with medium containing MTT, and the cells were incubated at 37℃ for 2 h. Cell viability was evaluated by measuring the absorbance of each well at 570 nm with a VersaMax™ microplate reader. Cell invasion assays were performed using a Transwell Boyden chamber (Corning) precoated with matrigel.

### Xenograft studies in nude mice

All procedures were approved by the Central South University Institutional Animal Use and Care Committee. All 6 to 8-week old male nude mice were subcutaneously injected into the flank with 1 × 10^6^ tumor cells (LNCaP sh-CTR, LNCaP sh-TOMM20; VCaP sh-CTR, VCaP sh-TOMM20) in 100 µl of a 1:1 solution of medium and Matrigel (Corning Biosciences). Eight weeks later, tumor formation rates were calculated for LNCaP cells. When the mean tumor volume of VCaP cells reached 100–150 mm^3^, mice were divided into four groups and received the following treatments for 3 continuous weeks: vehicle, Casodex (10 mg/kg/d, oral gavage). Tumor size and body weight were measured twice a week, and tumor xenograft volume (V) was calculated using the following formula: V = ab^2^/2 (a: the long diameter and b: the short diameter). The tumor xenografts were isolated at the endpoint of the experiment, and tumor size and weight was compared by statistical analysis.

### Metastasis studies in NOD/SCID mice via tail vein injection

To validate the impact of TOMM20 depletion on PCa metastasis, we firstly generated a PC3 luciferase cell line, and then was infected with lentiviruses expressing TOMM20-shRNA, or scramble control shRNA. The stable PC3 luciferase cells with TOMM20 knockdown were generated by drug selection. The cells were injected into NOD/SCID mice via tail veins (1.5 × 10^6^ cells/100 µL). The cancer metastasis was monitored by assessing the bioluminescence at a series of time points by a IVIS Imaging System (PerkinElmer). For the detection of bioluminescence, one mouse was intraperitoneally injected 200 µL of d-luciferin (15 mg/ml, Yeasen, Shanghai, China) before imaging. The photometry of the tumor was calculated by Living Image 3.1.0 software (Caliper Life Sciences), and the data were used to evaluate the degree of cancer metastasis. The mice survival rate was analyzed by using Kaplan–Meier survival curve and log-rank test.

### Human PCa tissues for immunohistochemical (IHC) analysis

Human benign prostatic hyperplasia (BPH) and PCa specimens were obtained from The Third Xiangya Hospital of Central South University, which is approved by Central South University Institutional Review Board. Specimens were obtained from the patients diagnosed with PCa (*n* = 39). Six BPH specimens were used as controls. Formalin-fixed, paraffin-embedded tissue slides were prepared. The expression of TOMM20 and AR was analyzed by IHC staining according to the manufacturer’s instructions (ZSGB-Bio, China).

### Sphere formation assays

Cells (5 × 10^2^) were seeded in 96-well ultra-low attachment culture plates (Corning Life Sciences) in MammoCult™ Basal Medium (Stemcell) with 4 µg/ml Heparin Solution (Stemcell) and 0.48 µg/ml Hydrocortisone Stock Solution (Stemcell) for 10–14 days. Spheres were counted under an inverted microscope in triplicate wells.

### Extraction of mitochondrial protein

Mitochondria were isolated from PCa cells and RWPE-1 cells using a mitochondria isolation kit (C3601, Beyotime Institute of Biotechnology, Nantong, Jiangsu, China) according to the manufacturer’s instructions. Briefly, cells were homogenized in ice-cold mitochondria isolation buffer with 1 mM PMSF and centrifuged at 600 g for 10 min at 4 °C. Subsequently, the supernatants were transferred to another centrifuge tube and centrifuged at 11,000 g for 10 min at 4 °C. The sediment was mitochondria. The remaining supernatants were centrifuged at 12,000 g for 10 min at 4 °C to obtain the cytoplasmic fraction. Protein samples were analyzed by SDS-PAGE.

### Measurement of intracellular ROS levels

Intracellular ROS levels were measured using a Reactive Oxygen Species Assay Kit (Beyotime Biotechnology, China) according to the manufacturer’s instructions. Briefly, stable cells expressing sh-TOMM20 and sh-Control were incubated with DCFH-DA at 37 °C for 20 min, and then were observed with a fluorescence microscopy (Olympus). The fluorescence was measured at 488 nm excitation and 525 nm emission by a fluorescence spectrophotometer (BD Bioscience).

### RNA-Seq and data analysis

Total RNA was extracted from LNCaP sh-CTR and LNCaP sh-TOMM20 cells using the TRIzol reagent according to the manufacturer’s protocol. RNA purity and quantification were determined using the NanoDrop 2000 spectrophotometer (Thermo Scientific, USA). RNA integrity was assessed using the Agilent 2100 Bioanalyzer (Agilent Technologies, Santa Clara, CA, USA). Then the libraries were constructed using TruSeq Stranded mRNA LT Sample Prep Kit (Illumina, San Diego, CA, USA) according to the manufacturer’s instructions. The transcriptome sequencing and analysis were conducted by OE Biotech Co., Ltd. (Shanghai, China). Reads per kilobase million (RPKM) for each gene were estimated. A gene is considered at a statistically significant level if the folds of expression difference were more than 1.5 between any two samples. The *p* value was calculated by Cufflinks. Bioinformatic analysis was performed using the OECloud tools at https://cloud.oebiotech.cn.

### Statistical analysis

All data were analyzed by GraphPad Prism 9.0 (GraphPad Software, San Diego, CA, USA; RRID:SCR_002798) or FlowJo software. Results are presented as mean ± SEM or mean ± SD as indicated. The statistical difference between two samples was analyzed by Students t test. One-way ANOVA was used to analyze the statistical difference of multiple groups. *P < 0.05 and **P < 0.01. *P* < 0.05 was considered as statistically significant.

## Results

### TOMM20 positively correlated with AR and the expressions decreased with the transdifferentiation to NEPC

**Fig. 1:**
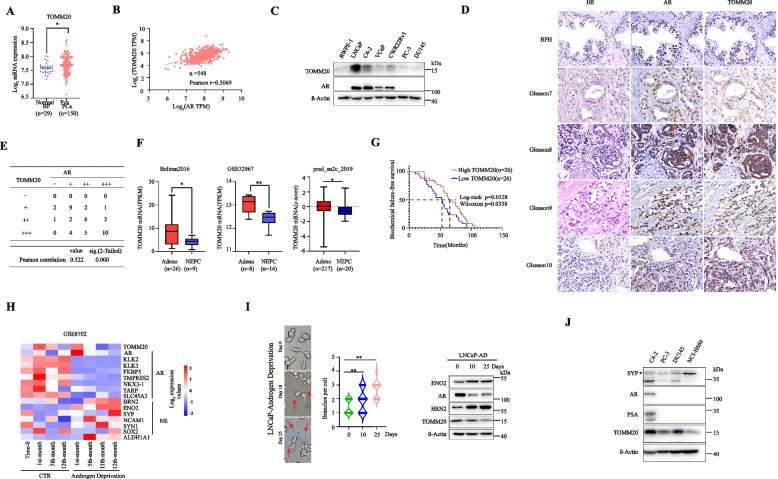
TOMM20 positively correlated with AR, and the expressions decreased with the transdifferentiation to NEPC. **A** The mRNA levels of *AR* and *TOMM20* genes in primary PCa specimens or the adjacent benign prostatic hyperplasia (BPH) tissues from a published RNA-seq dataset (GEO:GSE21034). **B** Correlation analysis of the mRNA levels of *AR* and *TOMM20* genes in PCa samples from TCGA datasets (http://vip.sangerbox.com/home.html). **C**. The protein level of AR or TOMM20 was assessed in non-malignant prostate epithelial cell line RWPE1 and PCa cells by western blotting. **D** AR and TOMM20 protein levels were assessed by IHC in human tumor specimens from 39 PCa and 6 BPH samples. **E** The protein levels of AR and TOMM20 in PCa specimens were quantified, and the correlation of two proteins was analyzed by Pearson’s correlation coefficient in SPSS software. **F** The mRNA levels of *TOMM20* gene in PCa (Adeno) and NEPC samples. The gene expression profiles derived from cBioPortal (Beltran, 2016, and prad_su2c_2019) and GEO dataset (GSE32967). **G**.Kaplan–Meier Biochemical failure-free survival analysis was performed for PCa patients co-treated with ADT and radiotherapy. The data was derived from a published dataset (GSE116918). **H** Heatmap of *TOMM20*, AR regulatory genes and NE markers in LNCaP cells after long-term androgen deprivation (GSE8702). **I** Representative images and neuritogenesis analysis of LNCaP cells at 10 or 25 days after androgen deprivation. Red arrowheads indicate neurite outgrowth. TOMM20, AR, BRN2 and ENO2 were assessed by western blotting. **J** The protein levels of SYP, AR, PSA or TOMM20 in PCa cell lines C4-2, PC-3, DU145 and NEPC cell line NCI-H660 were assessed by Western blotting

Aberrant overexpression of TOMM20 has been reported in several cancer types [27, 31–36]. By analyzing the GEO database(GSE21034 and GSE80609), we compared the mRNA levels of *TOMM20* gene between primary prostate cancer (PCa) and the adjacent benign prostate(BP) or hyperplasia(BPH). The mRNA levels of *TOMM20* gene are higher in PCa than BP (Fig. 1A and Fig.S 1A). Since AR is a key molecule for PCa development, we further tested the expression correlation of *AR* and *TOMM20* genes in PCa (the data were from http://vip.sangerbox.com/home.html). TOMM20 is positively correlated with AR at the mRNA levels in 548 PCa cases from TCGA database (Person *r* = 0.5069, Fig. 1B), and at the protein levels in non-malignant prostate epthelial cell line RWPE-1 and PCa cell lines as shown in Fig. 1C. By immunohistochemical (IHC) analysis, we further validated the correlation of AR and TOMM20 protein levels in 6 BPH and 39 PCa cases. AR protein co-localized with TOMM20 in the cytoplasm of PCa cells, and highly and positively correlated. With the elevation of Gleason grade, the protein levels of AR and TOMM20 elevated simultaneously (Fig. 1D, E and Fig.S 1B). These data suggested that in all stages of PCa, the expression level of TOMM20 is closely and positively correlated with AR.

As we knew, 10–17% PCa transformed to NEPC when PCa patients developed resistance to the second-generation AR antagonists. AR signaling reduced or lost and the neuronal-associated genes (such as BRN2, SYP, ENO2) elevated in NEPC tissues [25, 37]. We compared the mRNA levels of *TOMM20* gene in PCa(Adeno) to NEPC by analyzing datasets from GEO and cBioPortal. TOMM20 significantly decreased in NEPC (Fig. 1F). The advanced PCa patients received androgen-deprivation therapy(ADT) together with radical radiotherapy. The ten-year biochemical failure-free survival(BRFS) of PCa patients with high TOMM20 level is much greater than those with low level (Fig. 1G). We then analyzed another GEO dataset (GSE8702) in which LNCaP cells were cultured in androgen-free medium for 12 months, which was regarded as a typical approach to promote the transdifferentiation of NEPC [38, 39]. After prolonged androgen deprivation, TOMM20 and the androgen/AR-regulatory genes robustly decreased, while neuronal-associated genes significantly elevated (Fig. 1H). To further validate the results, we cultured LNCaP cells in androgen-free medium for 25 days, and observed that more cells gained neuron-like morphology with more branches. The levels of TOMM20 and AR gradually decreased with the extension of androgen deprivation, while BRN2 and ENO2 were further up-regulated (Fig. 1I). We then compared TOMM20 protein in PCa cell lines C4-2, PC3 and DU145 to the NEPC cell line NCI-H660. The data showed that the protein level of TOMM20 was much lower in NCI-H660 cells than other PCa cells (Fig. 1J). These data indicated that the loss of TOMM20 might play a key role in the transdifferentiation of PCa to NEPC.

### AR protein interacts with TOMM20, and AR antagonists decreased their protein interaction

**Fig. 2:**
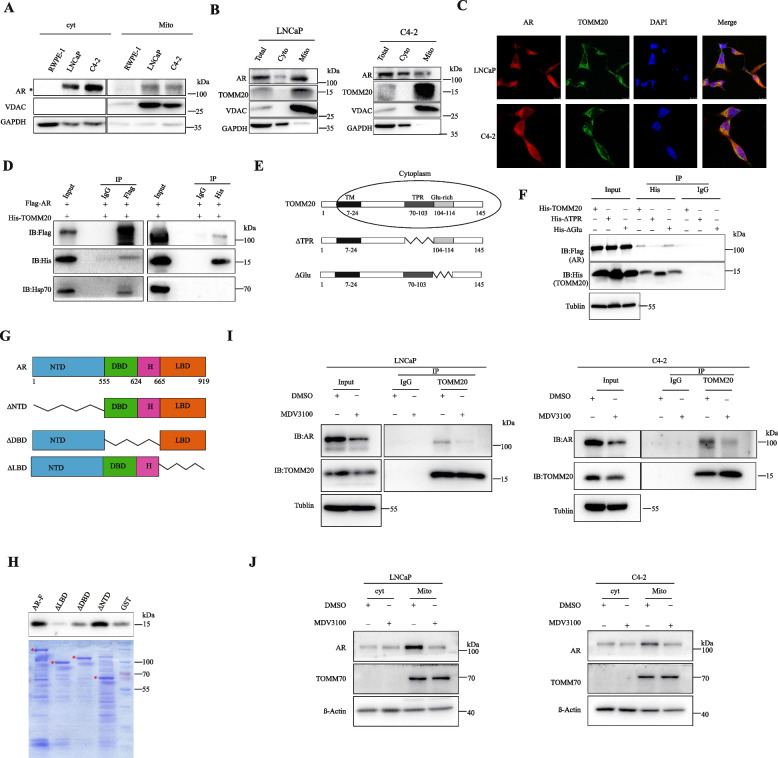
AR protein interacts with TOMM20, and AR antagonists inhibited their protein interaction. **A** and **B** Mitochondrial protein was separated from cytoplasmic protein of RWPE1 or PCa cells, and the level of AR or TOMM20 protein in the mitochondria (Mito) or in the cytoplasm without mitochondria (cyt) was assessed by western blotting, and VDAC was used as the specific marker of mitochondria. **C** AR or TOMM20 protein was labeled with fluorescent antibodies, and the co-localization of AR (red) and TOMM20 (green) was analyzed by confocol microscopy in LNCaP and C4-2 cells. **D** HEK293T cells were co-transfected with plasmids expressing Flag-AR or/and His-TOMM20, and the protein interaction of AR and TOMM20 was assessed by co-immunoprecipitation. **E** The schematic diagram of TOMM20 protein structure.** F**. HEK293T cells were co-transfected with plasmids expressing Flag-AR and His-TOMM20 wild type or mutants (His-TOMM20/His-∆TPR/His-∆Glu), and the protein interaction of AR and TOMM20 was assessed by co-immunoprecipitation.** G**. The schematic diagram of AR protein structure. **H** The interactive domain of AR with TOMM20 was identified by GST-pull down assay. The purified recombinant proteins of GST, GST-AR(FL), GST-AR(∆NTD), GST-AR(∆DBD), and GST-AR(∆LBD) were incubated with cell lysates in vitro as indicated, followed by immunoblotting with anti-TOMM20 antibody. **I** LNCaP and C4-2 cells were treated with MDV3100(20 µM) for 24 h, and then treated with 50 µM MG132 before protein extraction. The protein interaction of AR and TOMM20 was assessed by co-immunoprecipitation. **J** LNCaP and C4-2 cells were treated with MDV3100(20 µM) for 24 h, AR and TOMM70 expression in the mitochondria (Mito) or in the cytoplasm without mitochondria (cyt) was assessed by western blotting

It has been reported that AR is located in the mitochondria of PCa cells, and its expression is closely correlated with the malignancy [27]. To validate the cellular localization of AR, we first assessed AR protein levels inside (Mito) or outside (cyt) of the mitochondria in RWPE-1 and PCa cell lines LNCaP and C4-2. The results showed that PCa cells express more AR protein than RWPE-1, and AR is located in mitochondria of PCa cells in addition to cytoplasm (Fig. 2A). In LNCaP and C4-2 cells, AR protein is located in the mitochondria along with high levels of TOMM20 protein (Fig. 2B). Then, we stained AR and TOMM20 with fluorescent antibodies. AR protein clearly co-localized with TOMM20 (Fig. 2C).

We further studied whether AR protein physically interacts with TOMM20. The protein interaction of AR and TOMM20 was verified by co-immunoprecipitation (co-IP) of exogenously expressed proteins in HEK-293 T cells (Fig. 2D). TOMM20 consists of five conserved sub-domains: N-terminal membrane-anchor domain, linker domain rich in charged amino acids, tetratricopeptide repeat motif (TPR), glutamine-rich domain and C-terminal domain. In addition to the N-terminal membrane-anchor domain, the other four domains stay in the cytoplasm (Fig. 2E). We co-transfected the plasmids encoding AR or TOMM20 excluding the TPR/glutamine-rich domains to HEK-293 T cells, and assessed the interaction of exogenously expressed proteins by co-IP. The results identfied that AR interacts with the TPR domain of TOMM20 (Fig. 2F). Meanwhle, the AR proteins with individual domains (Fig. 2G) were mixed with cell lysates of LNCaP cells overexpressing TOMM20, and the protein domains of AR interacting with TOMM20 were identified by GST-pull down assay. The results showed that the LBD and Hinge domain of AR protein physically interact with TOMM20 (Fig. 2H).

The interaction of endogenously expressed AR and TOMM20 proteins was validated in both LNCaP and C4-2 cells in Fig. 2I (line1,3 and 5). Interestingly, AR antagonists such as MDV3100, significantly decreased the protein interaction of AR and TOMM20 (Fig. 2I,line 2,4 and 6). Beyond our expectations, MDV3100, in addition to inducing AR protein degradation(Fig.S 2,A and B), remarkably induced TOMM20 protein degradation(Fig. 2I), but had no impact on TOMM70, another member of translocase of outer mitochondrial membrane family (Fig. 2J). These results demonstrated that MDV3100-induced AR degradation specifically destroyed the protein stability of TOMM20.

### AR antagonists promoted TOMM20 protein degradation through autophagy-lysosomal pathway

**Fig. 3:**
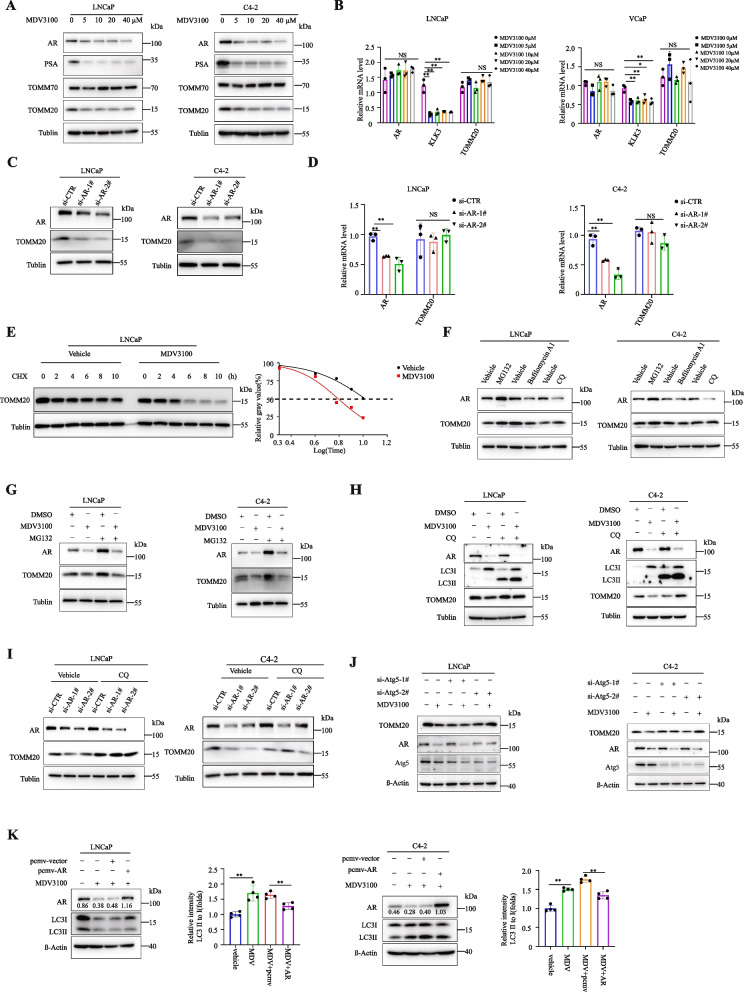
AR antagonists promote the protein degradation of TOMM20 through autophagy-lysosomal pathway. **A** LNCaP or C4-2 cells were treated with stepwise concentrations of MDV3100 for 24 h, and the protein levels of AR,PSA,TOMM70 and TOMM20 were assessed by western blotting. **B** LNCaP or VCaP cells were treated with stepwise concentrations of MDV3100 for 24 h, and the mRNA levels of *TOMM20*,AR and *KLK3* genes were assessed by RT-PCR. **C** LNCaP or C4-2 cells were transfected with siRNA against AR for 48 h, and the expression of TOMM20 protein was analyzed by western blotting. **D** LNCaP or C4-2 cells were treated with siRNA against AR for 48 h, and the mRNA level of *TOMM20* gene was analyzed by RT-PCR. **E** LNCaP cells were pre-treated with MDV3100 (40 µM) for 24 h, followed by treatment with CHX (50 µM) for 0, 2, 4, 6, 8 and 10 h. TOMM20 protein was analyzed by western blotting. **F** LNCaP or C4-2 cells were treated with MG132, Bafilomycin A1 or CQ for 18 h, and TOMM20 protein was assessed by western blot. **G** LNCaP or C4-2 cells were treated with MDV3100 (40 µM) for 24 h, and then treated with MG132 (50 µM) for an additional 18 h. AR or TOMM20 protein was assessed by western blotting. **H** LNCaP or C4-2 cells were treated with MDV3100 (40 µM) for 24 h, followed by CQ (25 µM) treatment for an additional 18 h. AR or TOMM20 protein was assessed by western blotting. **I** LNCaP or C4-2 cells were transfected with siRNA against AR for 48 h, and then treated with CQ (25 µM) for an additional 18 h. AR or TOMM20 protein was assessed by western blotting. **J** LNCaP or C4-2 cells were transfected with si-ATG5 for 48 h, followed by MDV3100 treatment for 24 h. AR or TOMM20 protein was assessed by western blotting. **K** LNCaP or C4-2 cells were transfected with pcmv-AR for 24 h, followed by MDV3100 treatment for 24 h. LC3II and LC3I were assessed by western blotting and the autophagy was assessed by measuring the ratio of LC3II/LC3I

We treated LNCaP or C4-2 cells with MDV3100 for 24 h, and found that MDV3100 significantly induced the protein degradation of TOMM20 in addition to AR, but had no impact on the mRNA levels of *TOMM20* and *AR* genes (Fig. 3A,B and Fig.S 3A). However, MDV3100 had no impact on TOMM20 protein stability in AR-negative PC-3 and DU145 cells (Fig.S 3B). The results indicated that AR antagonists induced TOMM20 protein degradation by destroying AR protein stability. Since AR protein interacts with TOMM20, and MDV3100 significantly reduced their interaction, we hypothesized that AR sustained TOMM20 protein stability. AR depletion with siRNA significantly decreased the protein level of TOMM20, but had no effect on the mRNA levels of *TOMM20* gene (Fig. 3C and D). We further tested the impact of AR antagonists on the half-life of TOMM20 protein in LNCaP cells. With the inhibition of new synthesized protein by CHX, MDV3100 significantly decreased the half-life of TOMM20 protein, which indicated that AR antagonists accelerated the protein degradation of TOMM20 (Fig. 3E). As we knew, the mechanism of intracellular protein degradation includes the ubiquitin–proteasome system (UPS) and autophagy-lysosomal pathway. We further investigated the regulatory mechanisms by which AR antagonists induced the protein degradation of TOMM20. First, we treated LNCaP or C4-2 cells with the USP inhibitor MG132 or autophagy inhibitors Bafilomycin A1 or Chloroquine (CQ), and identified the recycle mechanism of TOMM20 protein. The results showed that TOMM20 protein degradation mainly through UPS rather than autophagy under physiological conditions (Fig. 3F). Interestingly, the MDV3100-induced TOMM20 protein degradation might be reversed by autophagy inhibitor CQ, but not by UPS inhibitor MG132 (Fig. 3G,H and Fig.S 3C). These results were verified when AR was depleted by siRNA (Fig. 3I). To further verify that AR sustains the protein stability of TOMM20 by inhibiting the autophagy-mediated degradation pathway, we inhibited the autophagy pathway by depleting ATG5 in LNCaP and C4-2 cells, and then treated cells with MDV3100. The results demonstrated that autophagy inhibition significantly prevented MDV3100-induced TOMM20 protein degradation (Fig. 3J). In the effort to further validate the roles of AR in MDV3100-induced autophagy, we assessed the degree of autophagy (ratio of LC3-II/LC3-I) when AR was artificially overexpressed. The rescue of AR expression remarkably decreased the degree of MDV3100-induced autophagy (Fig. 3K). The results clarified that AR antagonists promoted TOMM20 protein degradation through autophagy-lysosomal pathway.

### TOMM20 protein degradation elevated the intracellular ROS level

**Fig. 4:**
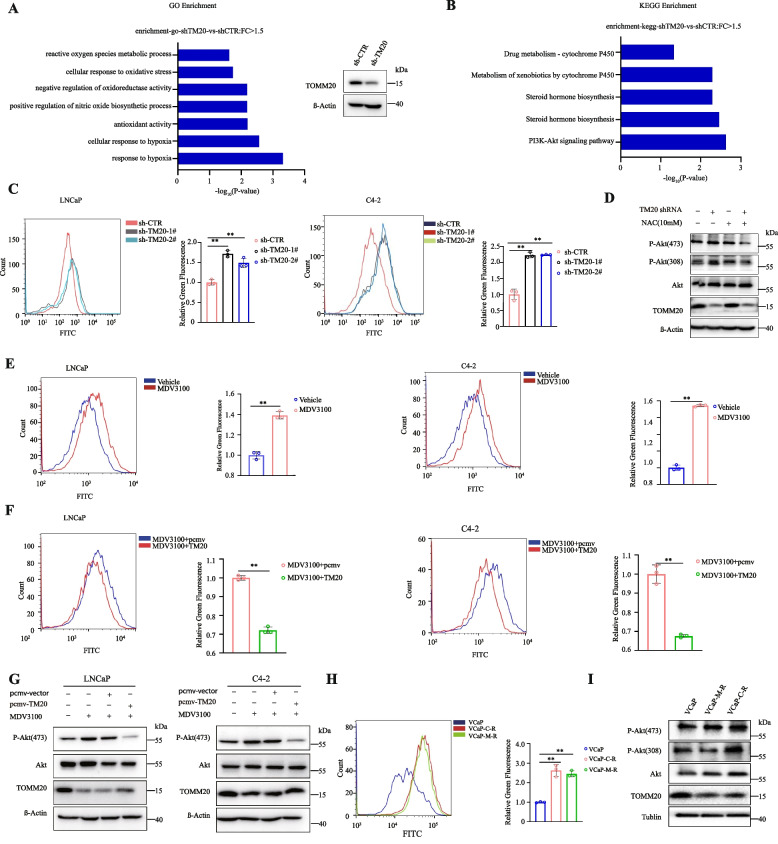
TOMM20 protein degradation elevated the intracellular ROS level. **A** Left:GO analysis was performed on all deregulated genes (DEGs) between sh-TOMM20 and sh-CTR. The blue bars of X-axis denote the − log_10_ values of *p* values on enriched GO terms, *p* value < 0.05. Y-axis represents the enriched GO terms of all DEGs between sh-TOMM20 and sh-CTR.Right:the protein expression of TOMM20 in RNA-seq samples. **B** KEGG pathway analysis was performed on all DEGs between sh-TOMM20 and sh-CTR. The blue bars of X-axis denote the − log_10_ values of *p* values on the enriched KEGG pathway categories, *p* value < 0.05. Y-axis represents the enriched KEGG pathway categories of all DEGs between sh-TOMM20 and sh-CTR. **C** LNCaP or C4-2 cells stably expressing TOMM20 shRNA were established, and the intracellular ROS levels were tested by flow cytometry. **D** VCaP cells stably expressing TOMM20 shRNA were established. The cells were treated with/without NAC (10 mM) for 2 h, and the expression levels of phospho-Akt (Ser473) or phospho-Akt (Thr308) were assessed by western blotting. **E** LNCaP or C4-2 cells were treated with MDV3100 (20 µM) for 24 h, and the intracellular ROS levels were detected by flow cytometry. **F** LNCaP or C4-2 cells overexpressing TOMM20 were treated with MDV3100 (20 µM) for 24 h, and the intracellular ROS levels were detected by flow cytometry. **G** LNCaP or C4-2 cells were transfected with pcmv-TOMM20 for 24 h, followed by MDV3100 treatment for 24 h. phospho-Akt(Ser473) and Akt were assessed by western blotting. **H** The AR antagonist-resistant VCaP cells VCaP-casodex-R(VCaP-C-R) and VCaP-MDV3100-R(VCaP-M-R) cells were established, and the intracellular ROS levels were determined by flow cytometry. **I** The expression of phospho-Akt(Ser473) or phospho-Akt(Thr308) in the AR antagonist-resistant or parental VCaP cells were assessed by western blotting

Since AR antagonists decreased TOMM20 protein levels but have no effect on the mRNA level, we want to know the roles of TOMM20 in the development of drug resistance to AR antagonists. We first analyzed the mRNA levels of *TOMM20* gene in three pairs of PCa cells sensitive or resistant to AR antagonists from GEO datasets (GSE847). The mRNA levels of *TOMM20* gene were lower in drug resistant PCa cells (Fig.S 4A). To explore the exact mechanism, we searched for the most affected pathways by RNA-seq analysis when TOMM20 was depleted with shRNAs in LNCaP cells. The expression levels of ROS-associated genes significantly elevated (Fig. 4A),, suggesting that TOMM20 depletion might elevate ROS levels. Flow cytometry and cellular fluorescence showed that the depletion of TOMM20 significantly elevated the intracellular ROS level in LNCaP and C4-2 cells (Fig. 4C and Fig.S 4B). It is well known that the elevation of ROS levels activates PI3K/AKT, a cell survival signaling cascade [40]. The KEGG pathway analysis showed the depletion of TOMM20 elevated the genes in the PI3K/AKT signaling pathway (Fig. 4B). As we expected, the depletion of TOMM20 remarkably elevated the levels of phosphorylated AKT(p-AKT) in VCaP cells. The antioxidant N-acetyl-L-cysteine (NAC), by decreasing the TOMM20-elevated ROS level, reversely decreased the levels of phosphorylated AKT(Fig. 4D). We further validated that AR antagonist-promoted TOMM20 protein degradation exerted the same effects as TOMM20 depletion. As shown in Fig. 4E and Supplemental Fig. 4C, MDV3100 significantly elevated the intracellular ROS level when it induced TOMM20 protein degradation. The rescue of TOMM20 reversed MDV3100-elevated ROS levels (Fig. 4F and Fig.S 4C), and reversed MDV3100-elevated levels of phosphorylated AKT (Fig. 4G). It has been reported that the activation of PI3K/AKT signaling pathways probably results in the development of drug resistance to AR antagonists [41]. We established two AR antagonist-resistant PCa cell lines, VCaP-Casodex-R (VCaP-C-R) and VCaP-MDV3100-R (VCaP-M-R) [42]. In comparison to the parental VCaP cells, the protein levels of TOMM20 significantly decreased, while the intracellular ROS level, together with the level of phosphorylated AKT markedly elevated in the drug resistant cells (Fig. 4H,I and Fig.S 4D). These results suggested that AR antagonist-induced TOMM20 protein degradation might induce drug resistance by elevating the intracellular ROS level and activating the PI3K/AKT signaling pathways.

### TOMM20 depletion promoted PCa cell proliferation and invasion as well as drug resistance to AR antagonists

**Fig. 5:**
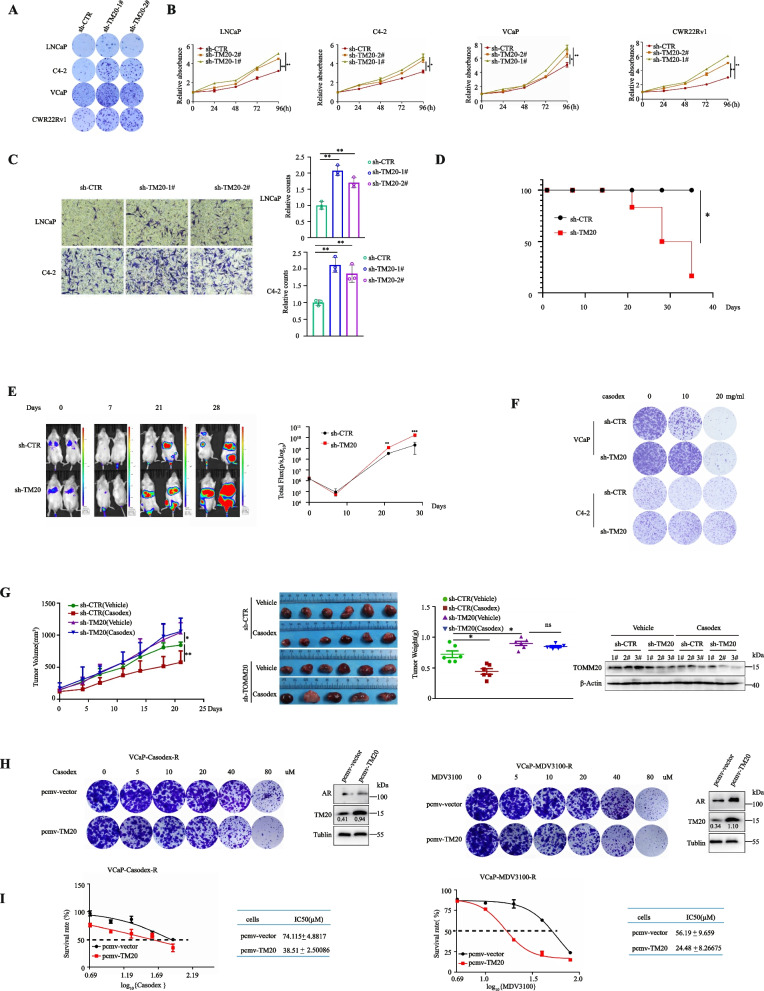
TOMM20 depletion promoted PCa cell proliferation and invasion and drug resistance to AR antagonists. **A** and **B** PCa cells stably expressing TOMM20 shRNA were established, and the effect of TOMM20 depletion on cell survival was tested by colony formation assays. Cell proliferation was measured by MTT assay. **C** LNCaP or C4-2 cells stably expressing TOMM20 shRNA were established, and the effect of TOMM20 depletion on cell invasion was measured by transwell assay. Relative invasion was quantified. **D** Kaplan–Meier survival curves of mice in the indicated experimental groups (*n* = 6/group). Statistical difference was analyzed by using the log-rank test. * *P* < 0.05. **E** TOMM20 knockdown promoted prostate cancer metastasis in vivo. Left: Representative bioluminescence images of NOD/SCID mice at different time points after tail vein injection of PC3 stable cells with TOMM20 knockdown or scrambled control. Right: Quantitative analysis of optical signals at the abdominal viscera area from bioluminescence (total flux, p/s) of the mice (*n* = 6 each group). T-test ***p < 0.001. **F** VCaP or C4-2 cells were treated with Casodex for 7 days, and then the cell colonies were stained with crystal violet. **G** VCaP cells stably expressing TOMM20 shRNA (sh-TM20) were used to induce tumor xenografts in immunodeficient nude mice, and then the mice were treated with Casodex. Tumor volumes were measured twice a week, and the growth curves of xenografts were drawn. The dissected tumors were weighed (*p < 0.05; **p < 0.01). TOMM20 depletion was verified by western blotting. **H** Casodex-resistant cells (VCaP-Casodex-R) and MDV3100 resistant cells (VCaP-MDV3100-R) were transiently transfected with TOMM20, and then treated with the stepwise concentrations of Casodex or MDV3100. Cell colonies were stained with crystal violet dye. TOMM20 overexpression was verified by western blotting. **I** Cell viability was measured by CCK8 assays. The assays were repeated three times, and the data were expressed as the Mean ± SD

We further investigated the effects of TOMM20 depletion on cell proliferation, using clonogenic and MTT assays. Consistently in LNCaP, C4-2, VCaP and CWR22Rv1 cell lines, TOMM20 depletion with shRNAs significantly accelerated cell proliferation (Fig. 5A,B). Using transwell-based assays, we found that TOMM20 depletion significantly promoted the invasion of LNCaP and C4-2 cells (Fig. 5C). We further performed the in vivo animal experiments to validate whether TOMM20 depletion promoted PCa metastasis. We firstly generated a stable PC-3 luciferase cell line with TOMM20-knockdown, and intravenously injected to NOD/SCID mice via tail veins. The non-targeting shRNA was used as the scrambled control. The cancer metastasis was monitored at a series of time points by a IVIS Imaging System. Consistent with the in vitro data, TOMM20 depletion significantly promoted the metastasis of prostate cancer to the abdominal viscera in NOD/SCID mice (Fig. 5E), and the survival rate of mice was remarkably reduced (Fig. 5D). We tested the impact of TOMM20 depletion on the sensitivity of PCa cells to AR antagonists. As shown in Fig. 5F and Supplemental Fig. 5A and 5B, TOMM20 depletion with shRNAs significantly enhanced drug resistance to Casodex in VCaP, C4-2 and LNCaP cells. We further examined the effects of TOMM20 depletion on the anti-tumor effect of Casodex in PCa xenografts. We first established a pair of VCaP cells stably expressing shTOMM20 or shCTR, and induced tumor xenografts in immune-deficient nude mice. The mice were treated with Casodex via o.p. for 21 days. The tumor growth curve and comparison of tumor sizes at the endpoint of the experiment demonstrated that TOMM20 depletion promoted tumor growth and reduced the anti-tumor effect of Casodex (Fig. 5G). Conversely, we tested whether the rescue of TOMM20 might improve the sensitivity of drug resistant PCa cells to AR antagonists Casodex and MDV3100. We elevated the levels of TOMM20 in the drug resistant VCaP cells by transient transfection, and tested the drug sensitivity to Casodex or MDV3100 by clonogenic formation assays. The results showed that the rescue of TOMM20 expression promoted drug sensitivity of the resistant PCa cells to Casodex or MDV3100 (Fig. 5H). The results were verified by CCK8 cell proliferation assay (Fig. 5I). The results demonstrated that TOMM20 depletion promoted cell proliferation and invasion, and further promoted the resistance to AR antagonists.

### TOMM20 knockdown promoted PCa cells to obtain the characteristics of cancer stem-like cells

**Fig. 6:**
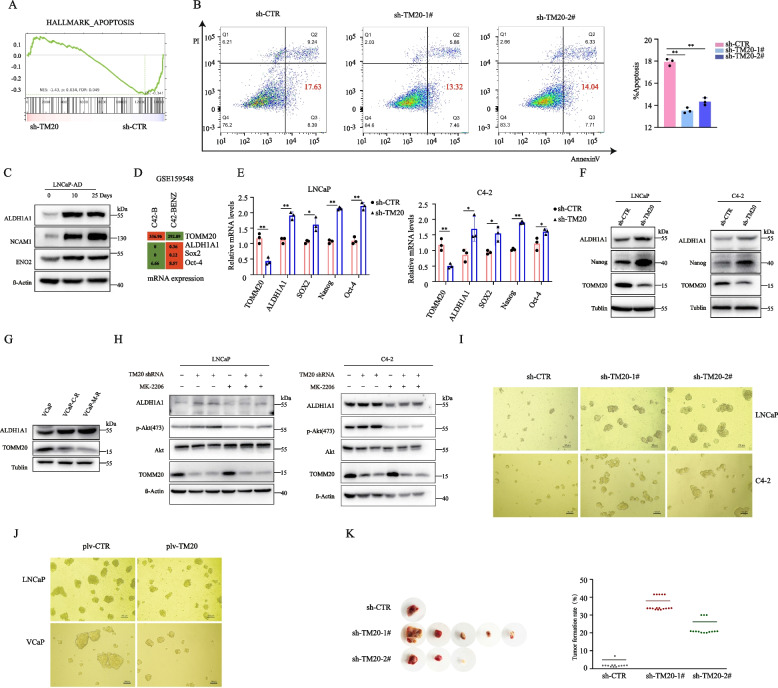
TOMM20 knockdown promoted PCa cells to obtain the characters of cancer stem-like cells. **A** Gene set enrichment analysis (GSEA) of the “Apoptosis” hallmark gene set in the RNA-seq datasets of LNCaP-shTOMM20 versus LNCaP-shCTR. **B** Anoikis resistance assay. LNCaP cells stably expressing TOMM20 shRNA (sh-TOMM20) were cultured on an ultra-low attachment plate for 3 days. The cells were stained by Annexin V/PI, and the apoptotic cells were analyzed by flow cytometry. **C**. ALDH1A1, NCAM1 and NSE were assessed by western blotting in the androgen-deprived LNCaP cells at 10 or 25 days. **D** Expression values of the indicated genes in enzalutamide-sensitive or resistant C4-2B cells from a published gene expression dataset (GEO: GSE159548). **E** and **F** LNCaP or C4-2 cells stably expressing TOMM20 shRNA (sh-TOMM20) were established, and the mRNA and protein levels of markers of cancer stem-like cells were measured by RT-qPCR or western blotting. **G** The protein levels of ALDH1A1 in AR antagonist-resistant or parental VCaP cells were assessed by western blotting. **H** TOMM20 stably depleted or parental LNCaP and C4-2 cells were treated with MK2206 (0.1 µM) for 48 h, and the cell lysates were subjected to immunoblotting. **I**. TOMM20 stably depleted or parental LNCaP or C4-2 cells were diluted and used for cell spheroid assays. **J** TOMM20 stably overexpressed or parental LNCaP or VCaP cells were diluted and used for cell spheroid assays. **K** TOMM20 stably depleted or parental LNCaP cells were used to induce tumor xenografts in immune-deficient nude mice. The xenograft tumors were harvested 8 weeks after cell incubation, and the incidence of tumor formation was calculated

Bioinformatics analysis of microarray gene expression revealed that the apoptosis-associated genes decreased in TOMM20 depleted cells (Fig. 6A). By performing an Anoikis assay, we found that TOMM20 stable depletion decreased cell apoptosis levels compared to the control cells (Fig. 6B). It has been reported that PCa stem cells (PCSC) play a critical role in the development of therapeutic resistance and subsequent cancer progression [43]. Since TOMM20 depletion remarkably promoted the resistance of PCa cells to AR antagonists, and decreased apoptosis when cultured in ultra-low attachment plates, we tested whether TOMM20 depletion promoted PCa cells to acquire the characteristics of PCSC. CSC markers ALDH1A1 elevated in LNCaP cells when cultured in androgen-free medium for 25 days (Fig. 6C). Gene expression analysis demonstrated the upregulation of CSC-associated genes concomitant with downregulation of TOMM20 in the ENZ-resistant C4-2B cells (Fig. 6D). The depletion of TOMM20 significantly elevated the mRNA or protein levels of such CSC markers as ALDH1A1, SOX2, Nanog and OCT-4 in LNCaP and C4-2 cells (Fig. 6E,F and Fig.S 6A). Compared to parental cells, TOMM20 level was lower but ALDH1A1 level was higher in the drug resistant VCaP cells (Fig. 6G). Since TOMM20 depletion elevated intracellular ROS levels and activated PI3K/AKT signaling pathway (Fig. 4D), we hypothesized that the acquisition of CSC characteristics resulted from AKT phosphorylation. Indeed, AKT inhibitor MK2206 remarkably decreased the level of ALDH1A1 (Fig. 6H). These data validated that TOMM20 degradation activated the PI3K/AKT pathways and promoted the acquisition of PCSC characteristics. We tested the effects of TOMM20 on the CSC characteristics using sphere formation assays. As shown in Fig. 6I and Supplemental Fig. 6B-C, TOMM20 depletion significantly increased the number and size of spheroids, while TOMM20 overexpression conversely decreased the number and size of spheroids in PCa cells (Fig. 6J). We tested the tumor formation capability of TOMM20-depleted LNCaP cells in immune-deficient nude mice. The ratio of LNCaP xenograft formation is ordinarily very low due to the specific characteristics of LNCaP cells. However, beyond our expectations, the ratio of xenograft formation significantly elevated from 1/12 to 5/12 for TOMM20-depleted LNCaP cells, and tumor sizes were remarkably increased (Fig. 6K). These data proved that TOMM20 depletion promoted the reprogramming of PCa cells to cancer stem-like cells.

### The expression of TOMM20 significantly decreased with transdifferentiation to NEPC and the development of drug resistance

**Fig. 7:**
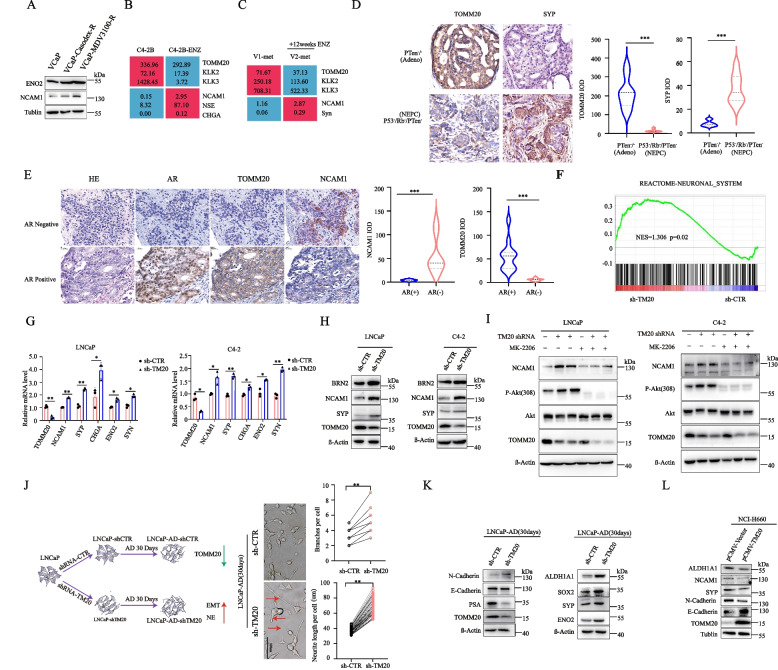
The expression of TOMM20 is significantly decreased with transdifferentiation to NEPC and the development of drug resistance. **A** Casodex-resistant or MDV3100-resistant VCaP cells were established, and the protein levels of ENO2 or NCAM1 were assessed by western blotting. **B** Expression values of the indicated genes in MDV3100-sensitive or resistant C4-2B cells from a published microarray gene expression dataset (GEO: GSE159548). **C** Expression values of the indicated genes from a published RNA-seq gene expression dataset (GEO: GSE70380). V1_met: rib metastasis was dagnosed at the first clinical visit before treatment. V2_met: metastasis was diagnosed at the second visit after enzalutamide (enza) treatment for 12 weeks. **D** Representative images of IHC staining for TOMM20 or SYP proteins in PCa tumor specimens of *Pten*^*−*^ (Adeo) or *PTen*^*−*^*/Rb*^*−*^*/P53*^*−*^(NEPC) mice (*n* = 3), and the quantitative data(IOD:integrated optical density) of IHC staining.for TOMM20. **E** Representative images of H.E. or IHC staining for TOMM20, AR or NCAM1 in PCa tumor specimens with(*n* = 4)/without(*n* = 4) AR expression,and the quantitative data of IHC staining for TOMM20 or NCAM1. **F** Biological pathways upregulated in TOMM20 stably depleted LNCaP cells. GSEA of “Neuronal_system” Reactome gene set in TOMM20 stably depleted versus parental LNCaP cells. **G** and **H** TOMM20 stably depleted or parental LNCaP or C4-2 cells were prepared, the mRNA levels of markers of NEPC were measured by RT-qPCR, and the protein levels of BRN2, NCAM1 or SYP were assessed by western blotting. **I**. The TOMM20 stably depleted or parental LNCaP and C4-2 cells were treated with AKT inhibitor MK2206 (0.1 µM) for 48 h, and the protein levels of NCAM1, phospho-AKT or AKT were assessed by western blotting. **J** and **K** Schema describing generation of LNCaP-AD-shTOMM20 and LNCaP-AD-sh-CTR cells by subjecting LNCaP-shTOMM20 or LNCaP-shCTR cells to androgen deprivation (AD) for 30 days. Representative images and neurogenesis analysis in LNCaP cells with or without TOMM20 depletion under AD. TOMM20, PSA, E-Cadherin, N-Cadherin, ENO2, Syp, SOX2 or ALDH1A1 were assessed by western blot. **L** The NEPC cells NCI-H660 were transfected with plasmids expressing TOMM20, the protein levels of ALDH1A1, NCAM1, SYP, N-Cadherin, E-Cadherin and TOMM20 were assessed by western blotting

Recent studies indicated that the transdifferentiation of adenocarcinoma cells into treatment-emergent NEPC (t-NEPC) is a critical mechanism of drug resistance after treatment with the second generation AR antagonists [17], and NEPC cells share the common characteristics of PCSCs [44]. We observed the characters of t-NEPC in two pairs of AR antagonist-resistant VCaP cells. In addition to the decrease of TOMM20 (Fig. 4I), the protein levels of typical NEPC markers ENO2 and NCAM1 remarkably elevated in Casodex or ENZ-resistant VCaP cells compared to parental cells (Fig. 7A). The results were validated by a RNA-seq dataset in a pair of parental and ENZ-resistant C4-2B cells(GSE159548). As shown in Fig. 7B, the mRNA levels of *TOMM20*, *KLK2* and *KLK3* significantly decreased, while the NEPC markers *NCAM1*, *ENO2* and *CHGA* significantly elevated in the ENZ-resistant cells. Interestingly, we observed similar alterations of gene expression in a CRPC and rib-metastasis patient who underwent ENZ treatment, and then developed drug resistance 12 weeks after treatment (PSA re-elevated from 17.7 ng/ml to 29.3 ng/ml) [45]. The RNA-seq data significantly decreased gene expression of *TOMM20*, *KLK2* and *KLK3,* while significantly elevated NEPC markers *NCAM1* and *SYN* when drug resistance developed ( Fig. 7C).

We further assessed the protein level of TOMM20 in PCa tumor specimens of *pten*^*−*^*/p53*^*−*^*/Rb1*^*−*^ or *pten*^*−*^ gene knockout mice by IHC analysis. The PCa tumors of *pten*^*−*^*/p53*^*−*^*/Rb1*^*−*^ gene knockout mice developed to NEPC phenotype [46], and TOMM20 expression significantly decreased in NEPC than PCa tumors of *pten-* gene knockout mice (Fig. 7D). Additionally, in the tumor specimens of advanced PCa patients, we observed low TOMM20 but high NCAM1 expression in the AR negatve PCa cells(Fig. 7E). RNA-seq data revealed that neuronal-associated genes were up-regulated in TOMM20-depleted cells (Fig. 7F and Fig.S 7A). TOMM20 depletion with shRNA significantly elevated the mRNA levels of *NCAM1*, *CHGA, EN02, SYP* and *SYN,* and elevated the protein levels of BRN2, NCAM1 and SYP in LNCaP and C4-2 cells (Fig. 7G and H).

We demonstrated that TOMM20 depletion elevated the intracellular ROS level and then activated the PI3K/AKT signaling pathway (Fig. 4C and D). TOMM20 depletion significantly elevated the levels of phosphorylated AKT and NCAM1 in LNCaP and C4-2 cells (Fig. 7I). However, the elevated levels NCAM1 were reversed by MK2206, an AKT phosphorylation inhibitor(Fig. 7I). These data validated that AKT phosphorylation is required for the NEPC-transdifferentiation of PCa.

We further validated that TOMM20 depletion promoted NEPC transdifferentiation. LNCaP cells stably expressing shTOMM20 were cultured in androgen-free medium for 30 days. Many cells showed the neuron-like morphology with more branches and longer antennae (Fig. 7J). Additionally, the protein expression of EMT, CSC and NEPC markers was obviously elevated, while the level of PSA decreased (Fig. 7K). Reversely, TOMM20 overexpressions in NEPC cell line NCI-H660 decreased the protein levels of ALDH1A1, SYP, NCAM1 and N-Cadherin, and elevated the level of E-Cadherin(Fig. 7L). The data validated that TOMM20 depletion promoted the NEPC transdifferentiaton of PCa.

## Discussion

Second-generation AR antagonists Enzalutamide and CYP17A inhibitor Arbiraterone have been approved for the treatment of mCRPC [47], and have greatly extended the lifespan and metastasis-free overall survival of patients. However, mCRPC patients inevitably develop acquired drug resistance. It has been proposed that treatment-emergent neuroendocrine PCa (t-NEPC) may be the critical mechanism of drug resistance [22]. Very few therapeutic options are available for patients at the t-NEPC stage in addition to platinum-based chemotherapy, and the median overall survival is less than one year [48]. The mechanisms of t-NEPC development need be explored, and specific diagnostic markers and effective therapeutic modalities are urgently required in the clinic.

As we know, the protein stability of cytoplasmic AR is maintained by forming a complex with chaperone proteins. HSP70 interacts with the N-terminal domain of AR, and HSP70 inhibitor induces AR protein degradation by inhibiting the binding of HSP70 to AR protein [5]. In the present study, we for the first time found that cytoplasmic AR protein interacts with the translocase of outer mitochondrial membrane 20 (TOMM20), and AR antagonists promoted the protein degradation of TOMM20 through autophagy-lysosomal pathway (Fig. 3H).

Mitochondria are ubiquitous multifunctional organelles with a double-membrane structure. TOMM20 is a conserved outer membrane protein. Nearly all precursors initially enter mitochondria by passing a common entry gate formed by the TOM complex, with TOMM20 acting as an initial docking site [49, 50]. TOMM20 depletion produces a large number of dysfunctional mitochondria with the loss of membrane potential [51]. Given the intrinsically complicated nature of mitochondria, mitochondrial dysfunction promotes ROS production and induces distinct stress signals [52–54]. In the present study, AR antagonists induced TOMM20 protein degradation, thereby elevating ROS level, and then activated the PI3K/AKT signaling pathway (Fig. 3A,E and Fig. 4C-E).

It is well known that activation of the PI3K/AKT signaling pathway is essential for maintenance of cancer stem-like cells (CSC) by regulating the CSC-specific transcription factor Nanog [55]. We for the first time reported a novel mechanism by which the autophagic degradation of TOMM20 resulted in drug resistance to AR antagonists by promoting the acquisition of cancer stem-like cell characteristics. TOMM20 depletion elevated the levels of the Yamanaka OSKM pluripotency reprogramming factors ALDH1A1, OCT-4, Nanog and SOX2, and promoted cell spheroid formation ability (Fig. 6E,F and I). Additionally, AKT phosphorylation is a typical characteristic of t-NEPC [56], and overactive AKT signaling has been identified in almost all advanced PCa, including NEPC [21]. TOMM20 depletion significantly up-regulated the levels of such NEPC markers NCAM1. The TOMM20 depletion-upregulated CSC and NEPC markers were remarkably reversed by the AKT phosphorylation inhibitor MK2206 (Fig. 6H and Fig. 7I). These results validated the activation of PI3K/AKT as one of the prerequisites for PCa cells to obtain CSC and t-NEPC characteristics.

t-NEPC is a critical mechanism of drug resistance to AR antagonists. AR antagonists induced TOMM20 autophagic degradation (Fig. 3H and J), which resulted in the acquisition of CSC characteristics (Fig. 6E and F)and NEPC transition (Fig. 7G and H). TOMM20 depletion promoted cell proliferation, EMT and cell invasion, and reduced drug sensitivity to AR antagonists (Fig. 5A-F). Conversely, the rescue of TOMM20 promoted the sensitivity of PCa cells to Enzalutamide and Bicalutamide (Fig. 5H and I). These data validated the roles of TOMM20 in drug sensitivity to AR antagonists. AR showed a high correlation with TOMM20 expression in the tumor specimens of PCa patients and PCa cells (Fig. 1C and D). The expression levels of TOMM20 significantly decreased in PCa patients with lost AR and NE marker overexpression (Fig. 7E), which suggested the critical roles of TOMM20 in NEPC transition and drug sensitivity.

**Fig. 8:**
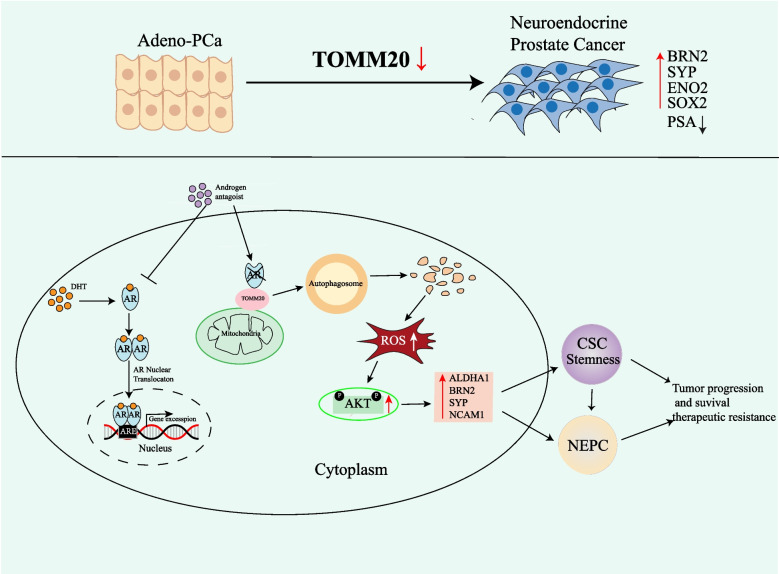
Schematic representation of TOMM20 degradation-promoted transdifferentiation of prostate adenocarcinoma(ADPC) to neuroendocrine prostate cancer (NEPC). AR antagonists promoted TOMM20 protein degradation through autophagy-lysosomal pathway, resulting in the elevation of intracellular ROS level and AKT phosphorylation. AKT phosphorylation then upregulated the levels of ALDH1A1, BRN2, NCAM1 or SYP. ADPC cells acquired the characters of cancer stem-like cells (CSC) and NEPC, and developed therapeutic resistance

Altogether, we identified for the first time the driver roles of TOMM20 protein degradation in transdifferentiation to NEPC, and clarified a novel molecular mechanism by which AR antagonists develop drug resistance (Fig. 8). The loss of TOMM20 in PCa tumor specimens might become a useful predictor of PCa sensitivity to AR antagonists.

### Supplementary Information

**Additional file 1: Supplementary Fig. 1.** A The mRNA levels of AR or TOMM20 gene in benign prostate hyperplasia, primary prostate cancer(CaP) and castration-refractory prostate cancer(CRPC) from a published dataset(GSEA80609). B. The levels of AR and TOMM20 in tumor specimens were quantified.

**Additional file 2: Supplementary Fig. 2.** A LNCaP cells were pre-treated with MDV3100 (40 µM) for 24h, and followed by treatment with CHX (50µM) for 0, 2, 4, 6, 8 and 10 hours. AR protein was analyzed by western blotting. B. C4-2 cells were pre-treated with MDV3100 (40 µM) for 24h, followed by treatment with CHX (50µM) for 0, 2, 4, 6, 8 and 10 hours. AR protein was assessed by western blotting.

**Additional file 3: Supplementary Fig. 3.** A VCaP cells were treated with stepwise concentrations of MDV3100 for 24h, and the protein levels of AR,TOMM20 and TOMM70 were assessed by western blotting. B. PC-3 or DU145 cells were treated with stepwise concentrations of MDV3100 for 24h, and the protein levels of TOMM70 and TOMM20 were assessed by western blotting. C. LNCaP or C4-2 cells were treated with MDV3100 (40µM) for 24h, followed by Bafilomycin A1(0.5 µM) treatment for additional 18h. AR and TOMM20 protein was assessed by western blotting.

**Additional file 4: Supplementary Fig. 4.** A Gene expression analysis of TOMM20 gene in three pairs of antiandrogen drug- resistant and parental PCa cells from GEO datasets (GSE847). B. LNCaP or C4-2 cells stably expressing TOMM20 shRNA were established, and the intracellular ROS levels were measured by fluorescence microscopy. C. LNCaP or C4-2 cells overexpressing TOMM20 were treated with MDV3100 (20µM) for 24h, and then intracellular ROS levels were measured by fluorescence microscopy. The overexpression of TOMM20 was validated by western blotting. D. AR antagonist-resistant VCaP cells VCaP-casodex-R and VCaP-MDV3100-R were established, and the intracellular ROS levels were measured by fluorescence microscopy.

**Additional file 5: Supplementary Fig. 5.** A LNCaP-shCTR or LNCaP-shTOMM20 cells were treated with Casodex at the indicated concentrations for 72h, and then the cell colonies were stained with crystal violet. B. C4-2-shCTR or C4-2-shTOMM20 cells were treated with Casodex at the indicated concentrations for 72h, and then the cell colonies were stained with crystal violet.

**Additional file 6: Supplementary Fig. 6.** A VCaP cells stably expressing TOMM20 shRNA (shTOMM20) were established, and the protein levels of markers of cancer stem-like cells were assessed by western blotting. B. TOMM20 stably-depleted or parental CWR22Rv1 cells were diluted for cell spheroid assays. C. TOMM20 stably-depleted or parental VCaP cells were cultured in soft agar for two weeks, and cell morphology was observed by microscopy.

**Additional file 7: Supplementary Fig. 7.** A GO analysis of RNA-seq data revealed that the neuronal-associated genes were up-regulated in TOMM20-depleted(sh-TM20) cells.

**Additional file 8: Supplementary Table. 1.** Sequences of siRNA and RT-PCR primers used in this study.

## Data Availability

The RNA-seq data in this manuscript have been submitted to the National Centre for Biotechnology (BioProject ID:PRJAN783133).The public datasets used in this study were downloaded from the Gene Expression Omnibus database (https://www.ncbi.nlm.nih.gov/geo/) and cBioPortal (http://www.cbioportal.org/). All relevant data are available from the corresponding authors upon reasonable request.
