# Supplementary figures and images for: AR antagonists develop drug resistance through TOMM20 autophagic degradation-promoted transformation to neuroendocrine prostate cancer
Source: J Exp Clin Cancer Res. 2023 Aug 10;42:204. doi: 10.1186/s13046-023-02776-0 (PMC10413764; doi:10.1186/s13046-023-02776-0)

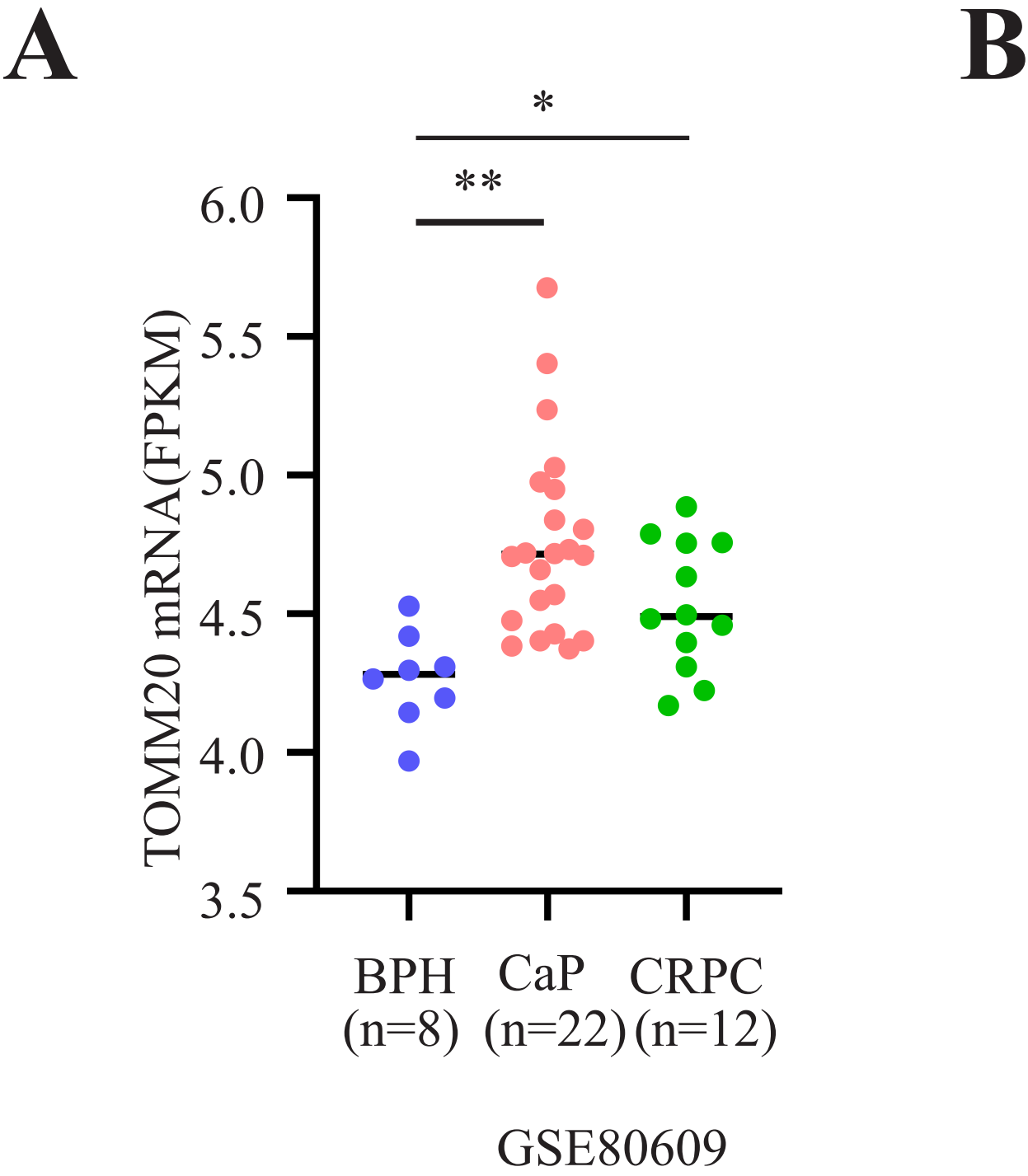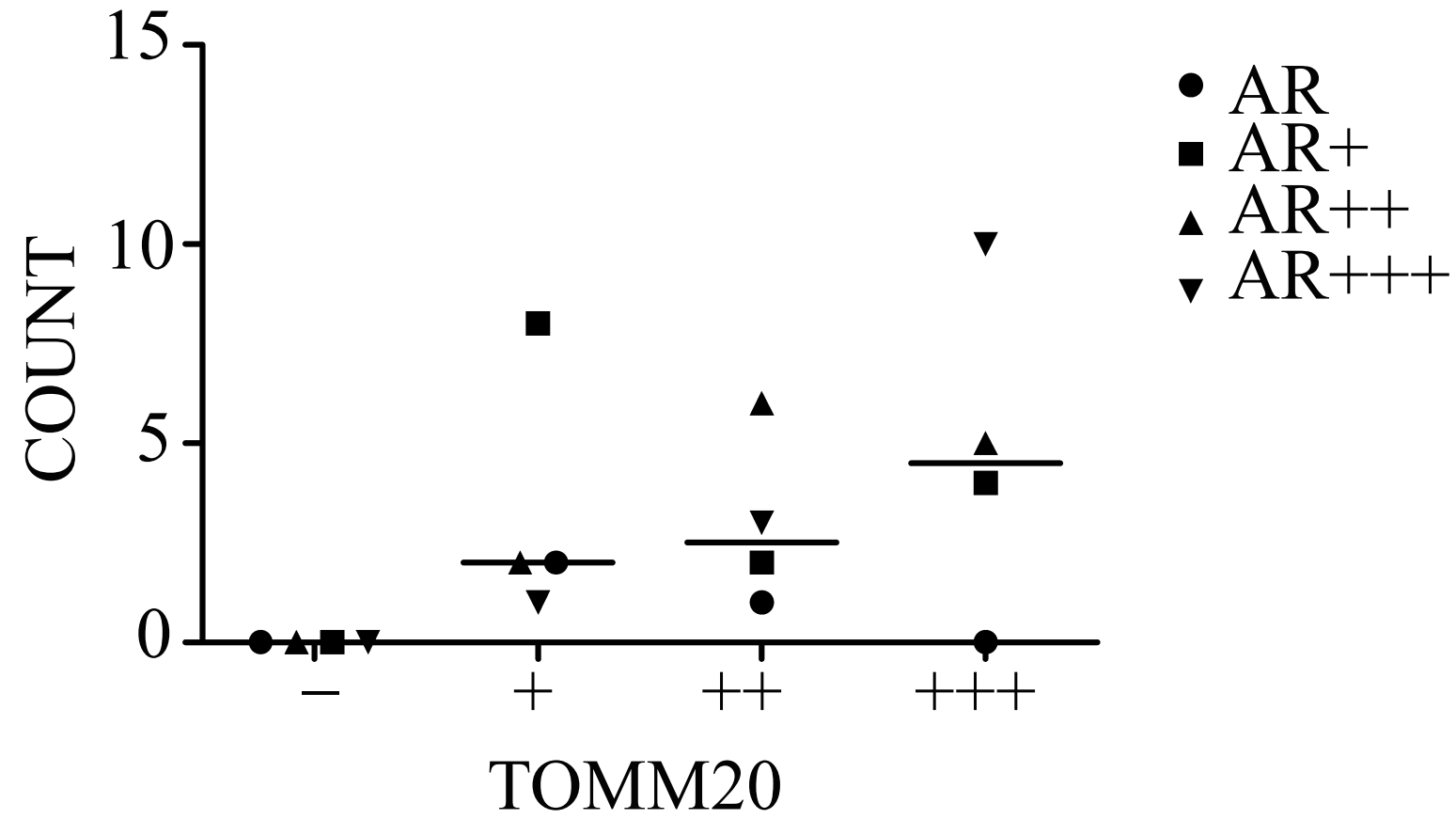

Supplement: Supplementary file 1 — Additional file 1: Supplementary Fig. 1. A The mRNA levels of AR or TOMM20 gene in benign prostate hyperplasia, primary prostate cancer(CaP) and castration-refractory prostate cancer(CRPC) from a published dataset(GSEA80609). B. The levels of AR and TOMM20 in tumor specimens were quantified. [file 13046_2023_2776_MOESM1_ESM.pdf]

**A**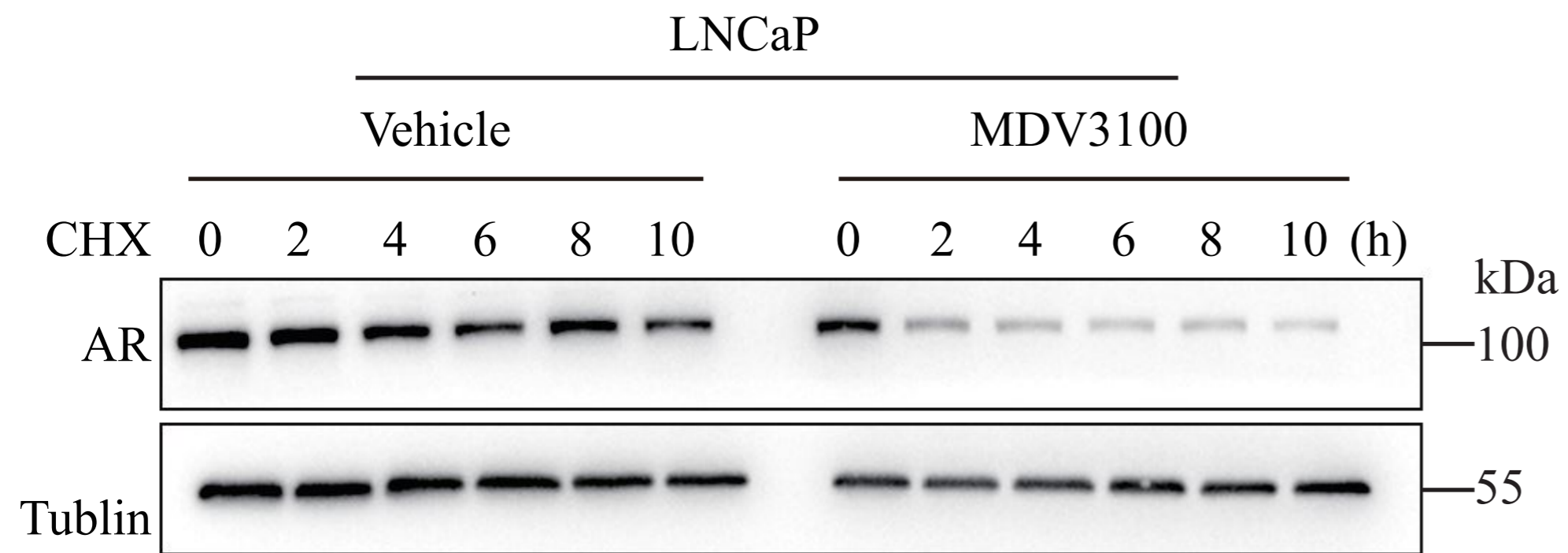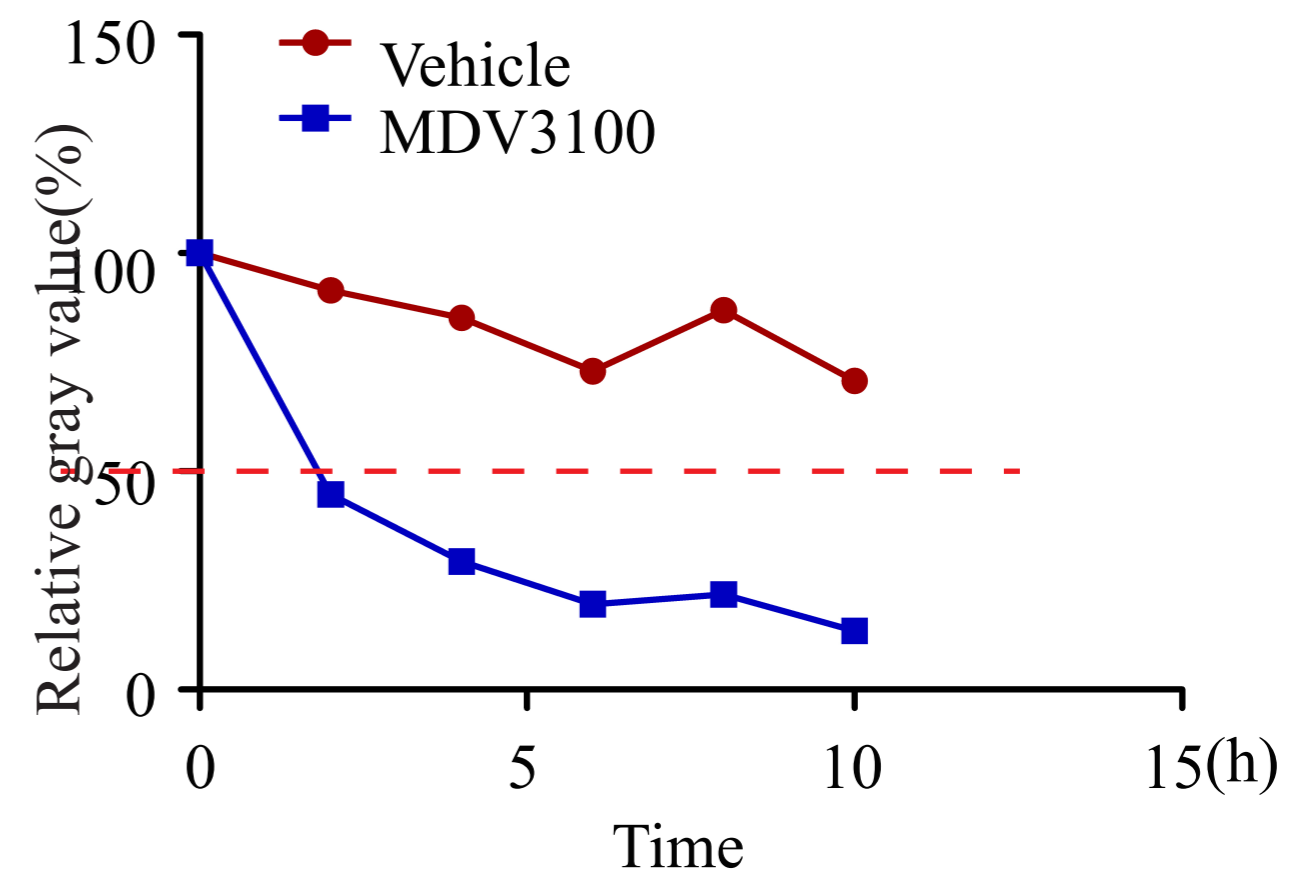**B**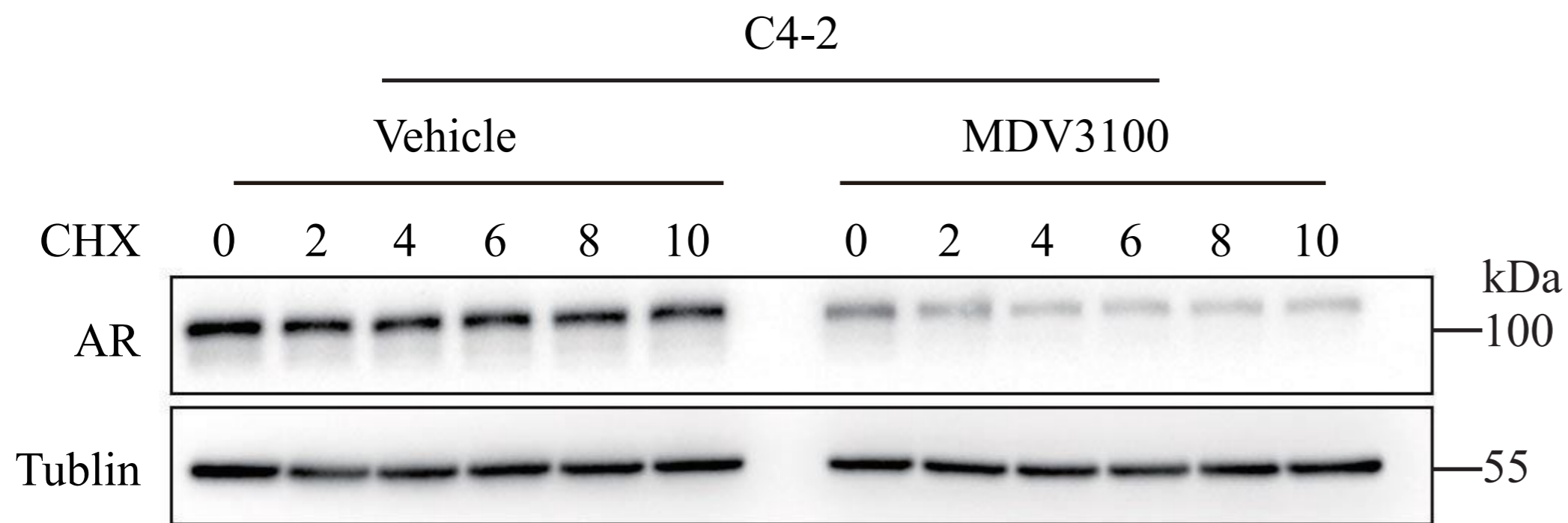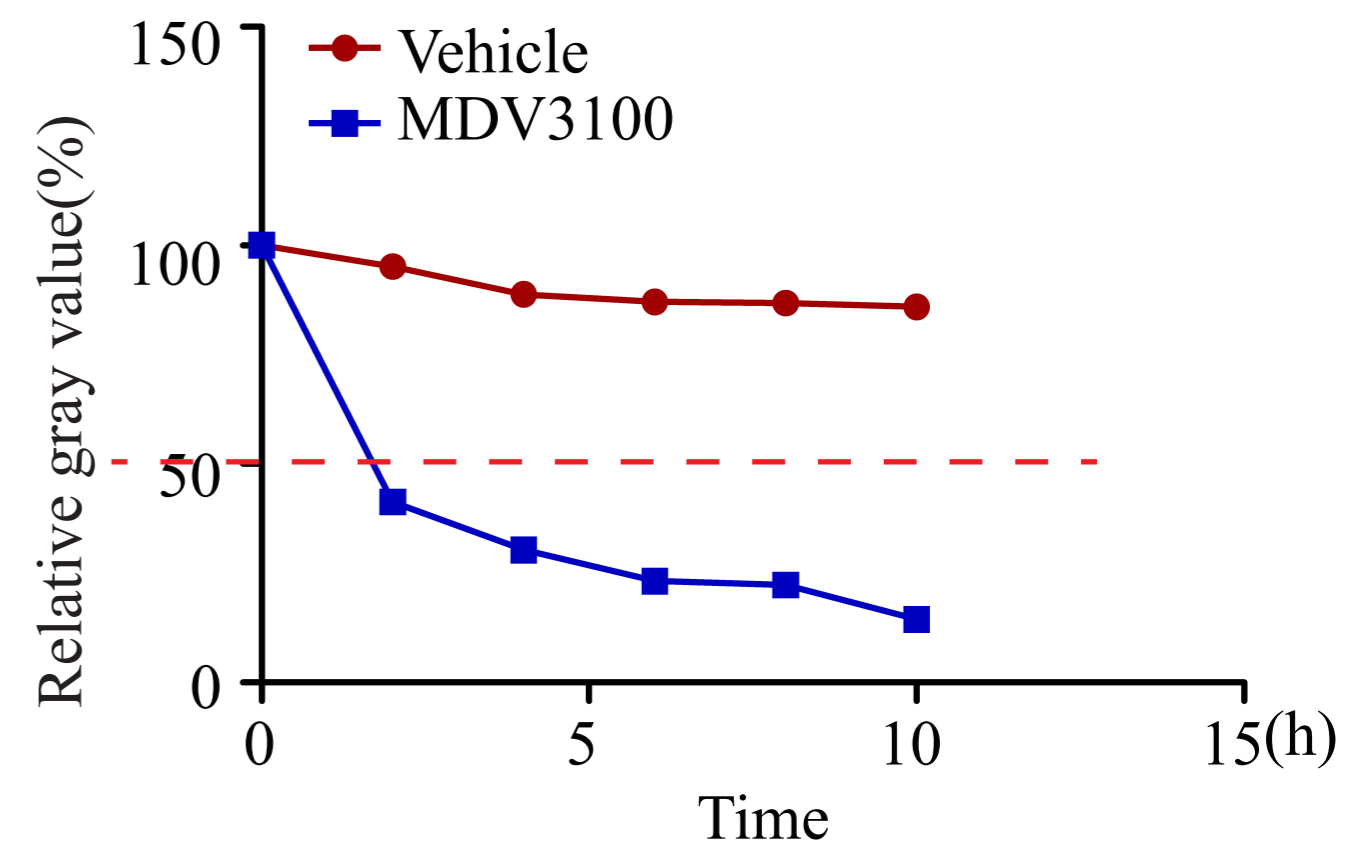

Supplement: Supplementary file 2 — Additional file 2: Supplementary Fig. 2. A LNCaP cells were pre-treated with MDV3100 (40 µM) for 24h, and followed by treatment with CHX (50µM) for 0, 2, 4, 6, 8 and 10 hours. AR protein was analyzed by western blotting. B. C4-2 cells were pre-treated with MDV3100 (40 µM) for 24h, followed by treatment with CHX (50µM) for 0, 2, 4, 6, 8 and 10 hours. AR protein was assessed by western blotting. [file 13046_2023_2776_MOESM2_ESM.pdf]

**A**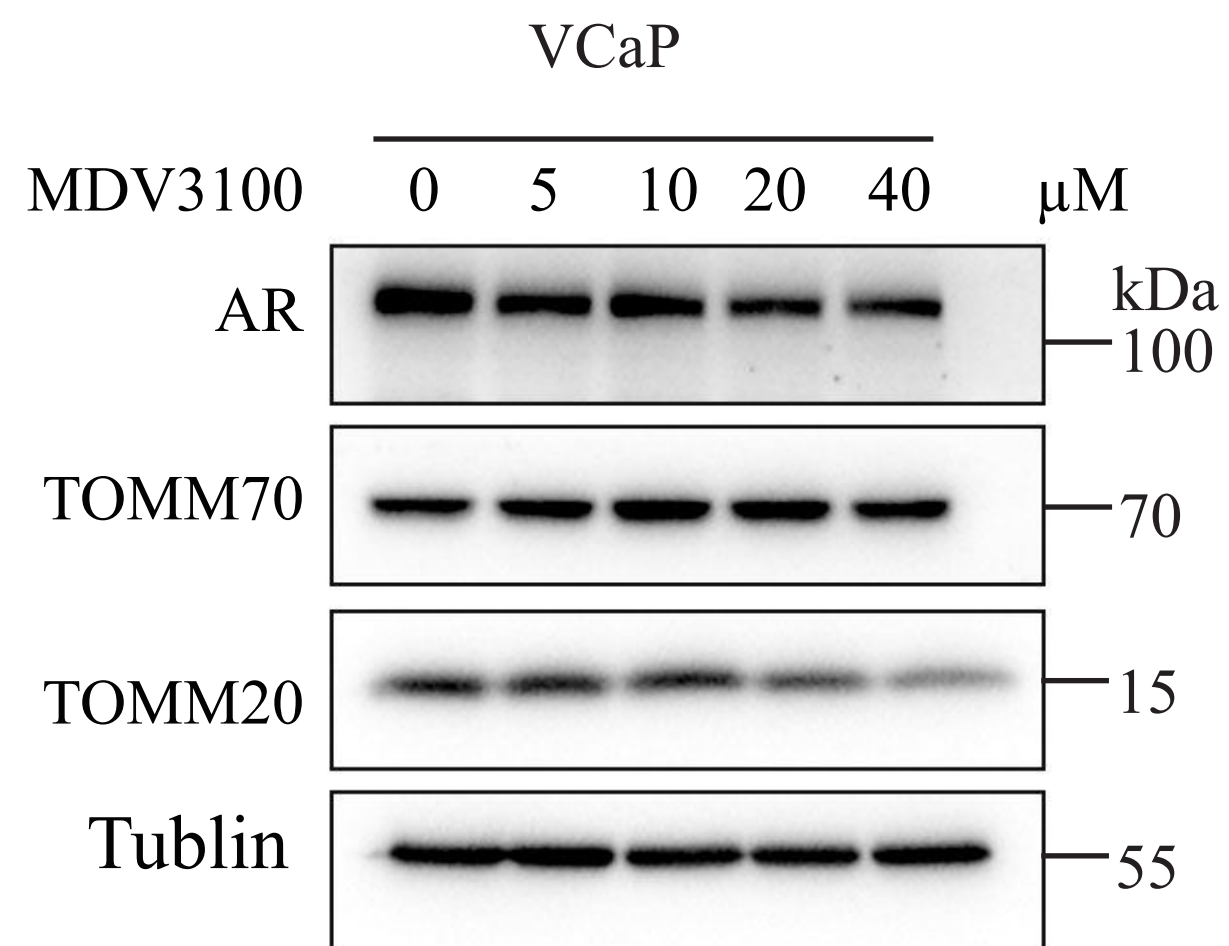**B**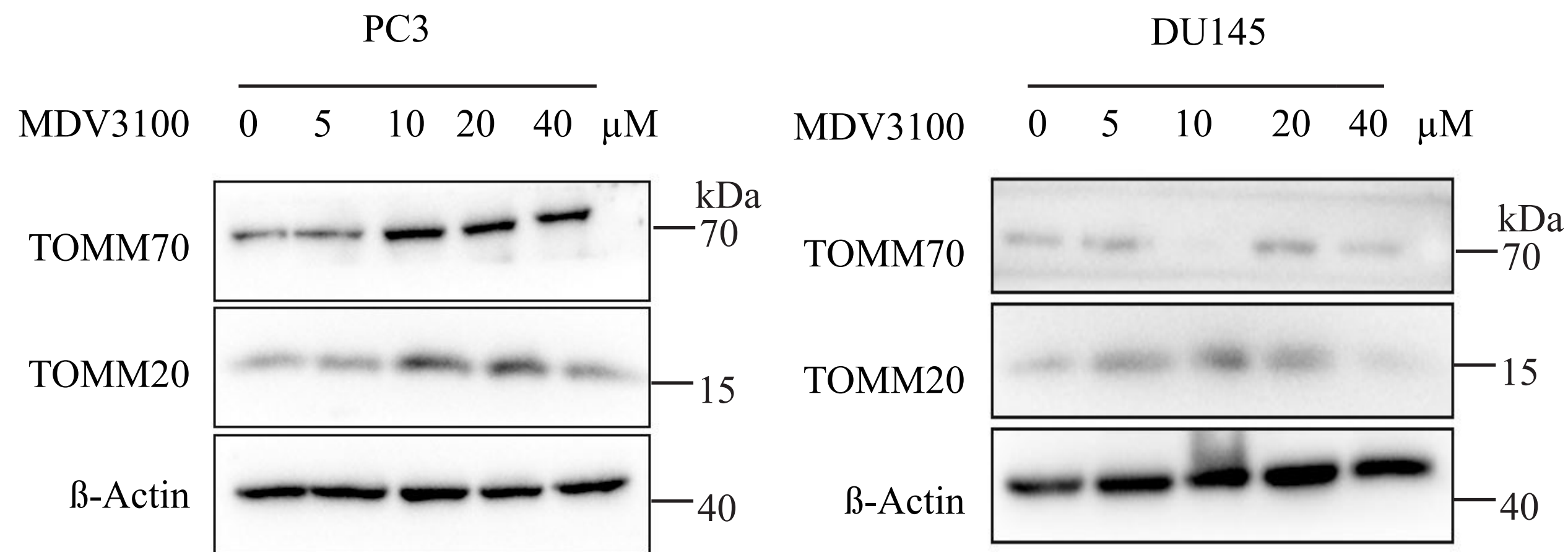**C**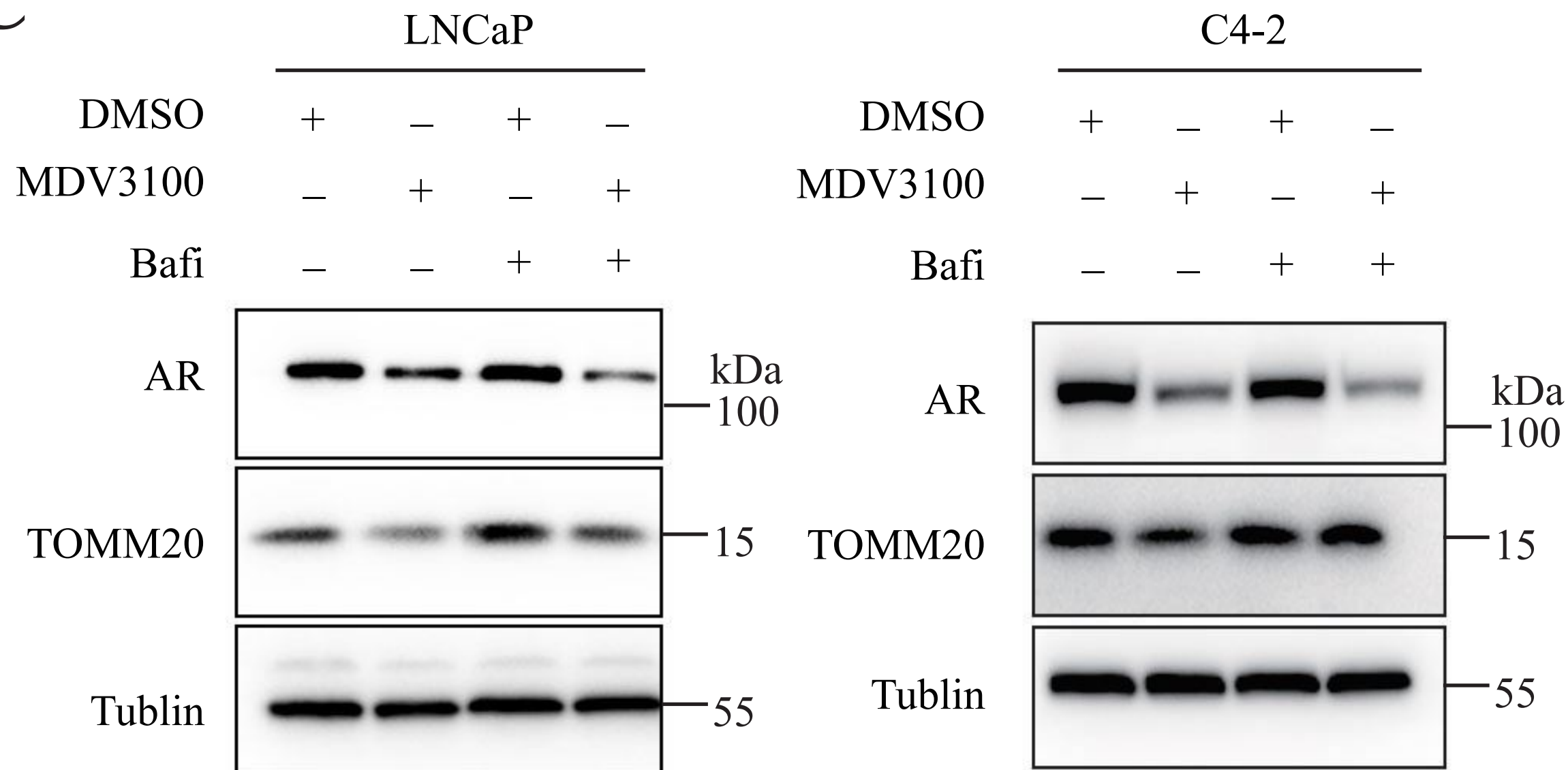

Supplement: Supplementary file 3 — Additional file 3: Supplementary Fig. 3. A VCaP cells were treated with stepwise concentrations of MDV3100 for 24h, and the protein levels of AR,TOMM20 and TOMM70 were assessed by western blotting. B. PC-3 or DU145 cells were treated with stepwise concentrations of MDV3100 for 24h, and the protein levels of TOMM70 and TOMM20 were assessed by western blotting. C. LNCaP or C4-2 cells were treated with MDV3100 (40µM) for 24h, followed by Bafilomycin A1(0.5 µM) treatment for additional 18h. AR and TOMM20 protein was assessed by western blotting. [file 13046_2023_2776_MOESM3_ESM.pdf]

**A**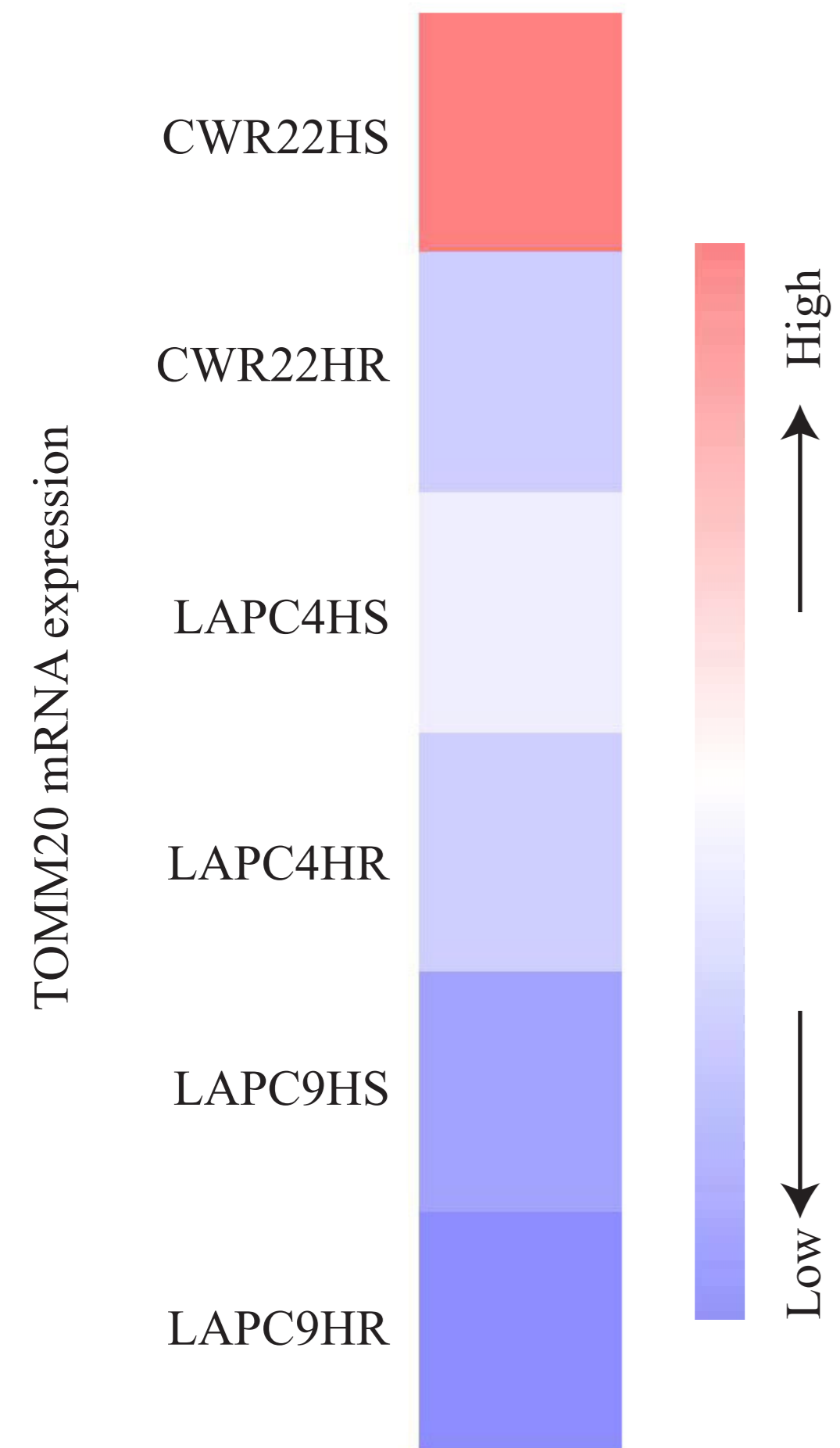**C**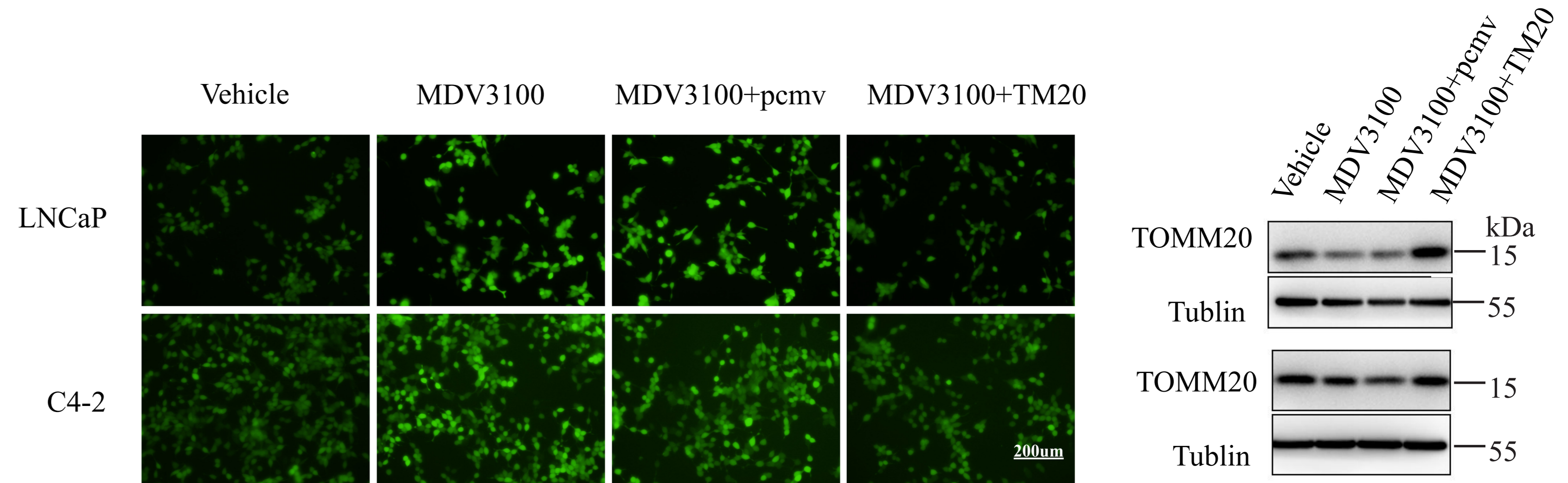**B**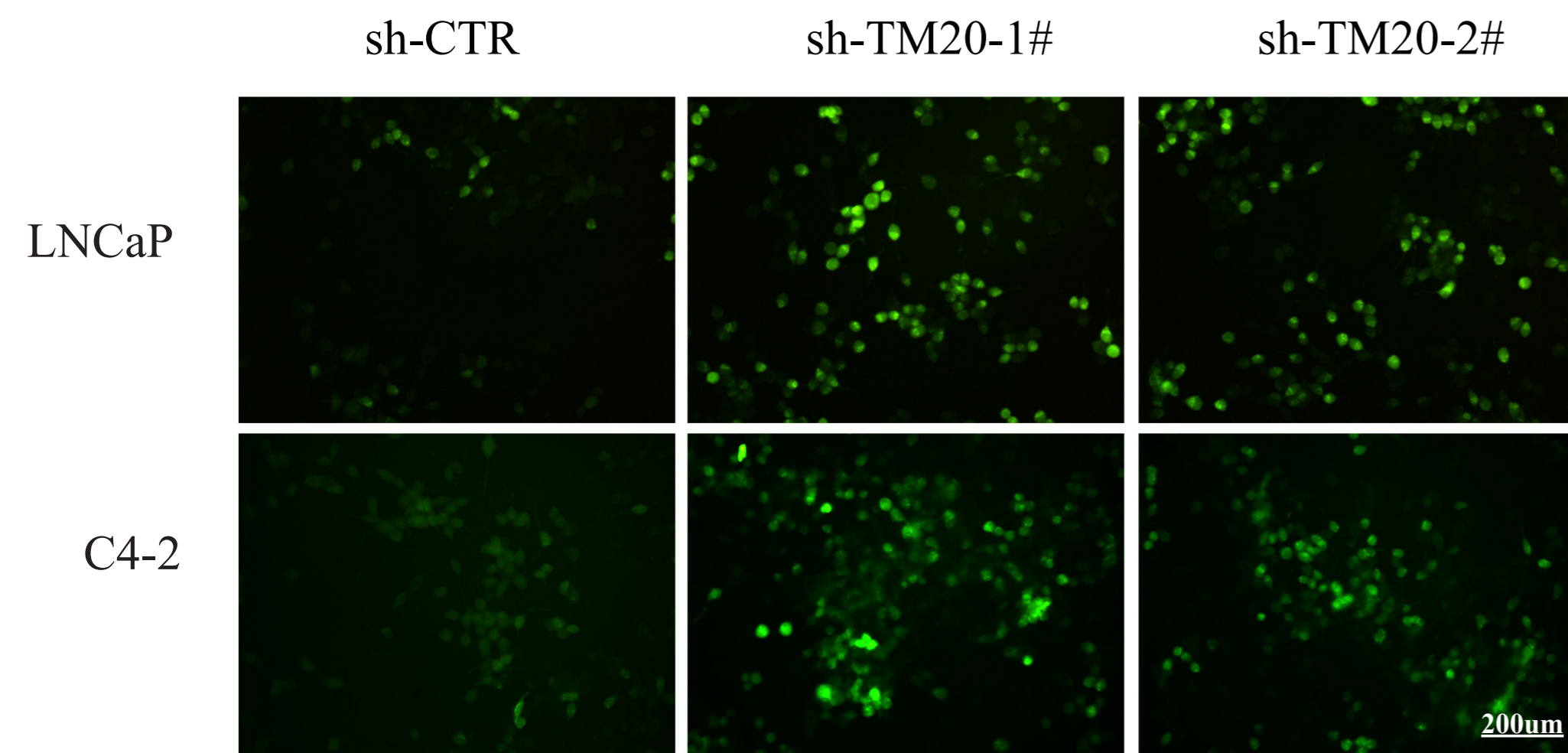**D**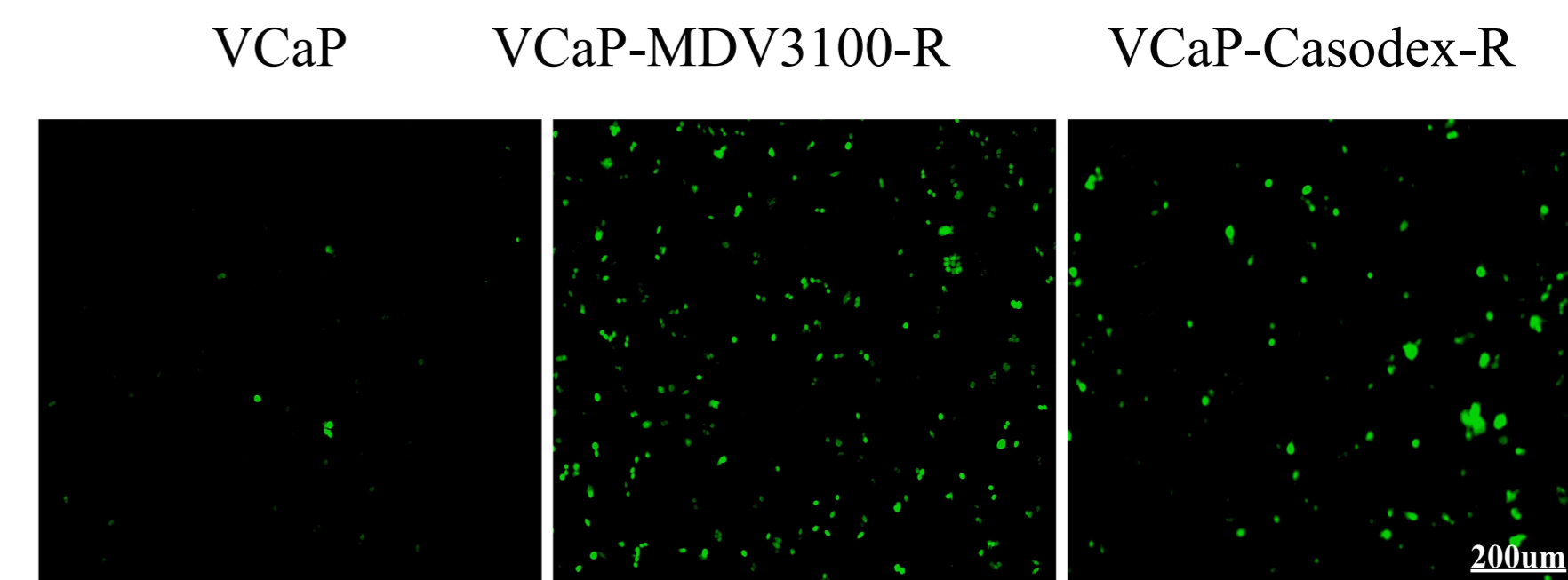

Supplement: Supplementary file 4 — Additional file 4: Supplementary Fig. 4. A Gene expression analysis of TOMM20 gene in three pairs of antiandrogen drug- resistant and parental PCa cells from GEO datasets (GSE847). B. LNCaP or C4-2 cells stably expressing TOMM20 shRNA were established, and the intracellular ROS levels were measured by fluorescence microscopy. C. LNCaP or C4-2 cells overexpressing TOMM20 were treated with MDV3100 (20µM) for 24h, and then intracellular ROS levels were measured by fluorescence microscopy. The overexpression of TOMM20 was validated by western blotting. D. AR antagonist-resistant VCaP cells VCaP-casodex-R and VCaP-MDV3100-R were established, and the intracellular ROS levels were measured by fluorescence microscopy. [file 13046_2023_2776_MOESM4_ESM.pdf]

**A**

LNCaP

Casodex      0      6.25      12.5      25      50      100     $\mu\text{M}$

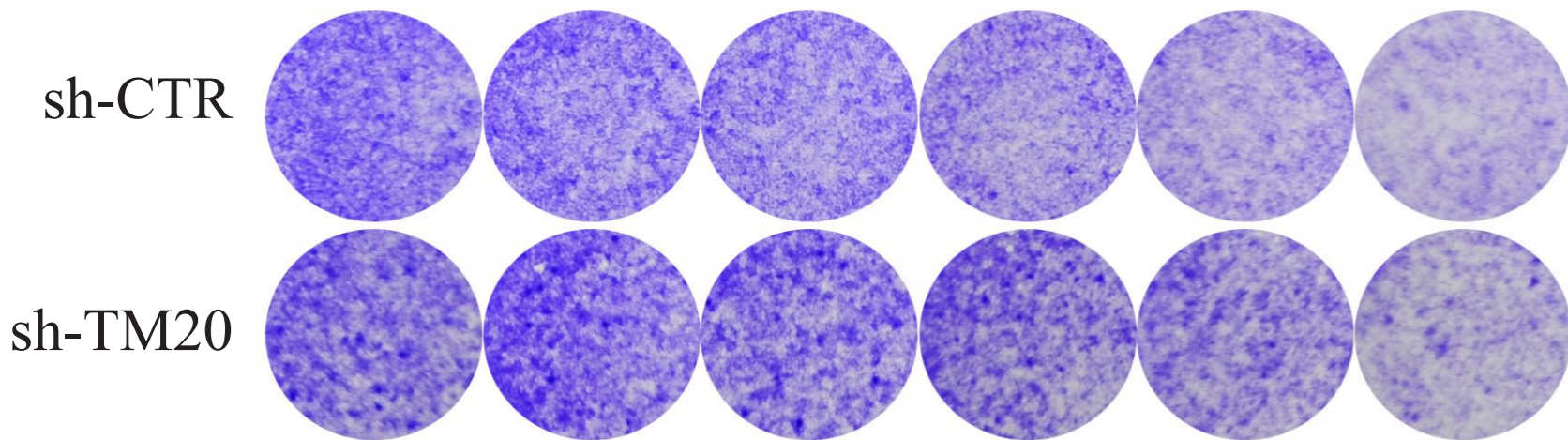**B**

C4-2

Casodex      0      6.25      12.5      25      50      100     $\mu\text{M}$

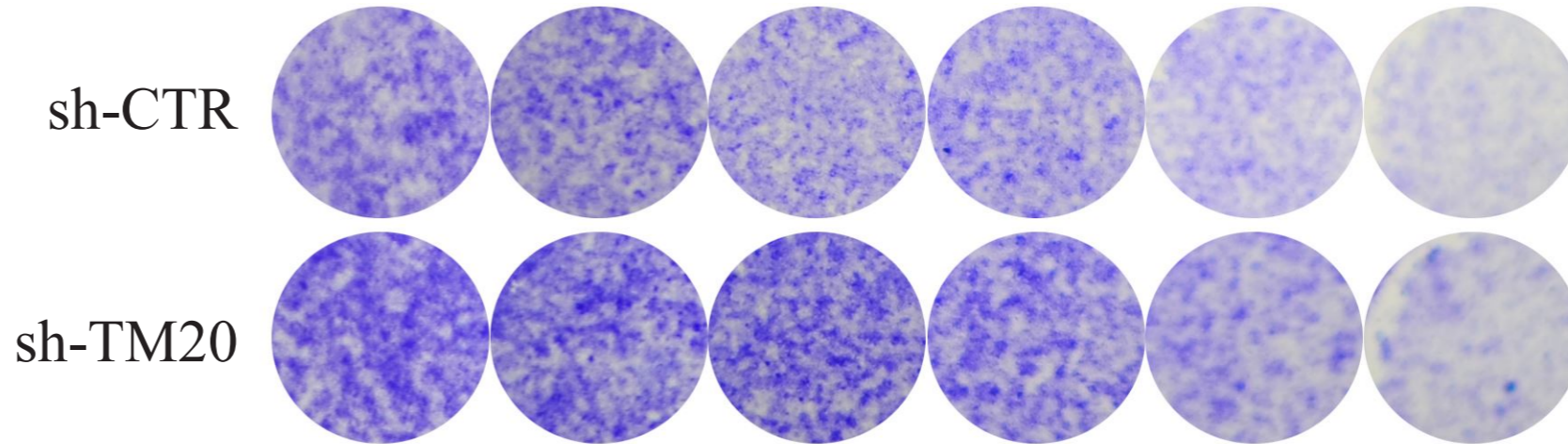

Supplement: Supplementary file 5 — Additional file 5: Supplementary Fig. 5. A LNCaP-shCTR or LNCaP-shTOMM20 cells were treated with Casodex at the indicated concentrations for 72h, and then the cell colonies were stained with crystal violet. B. C4-2-shCTR or C4-2-shTOMM20 cells were treated with Casodex at the indicated concentrations for 72h, and then the cell colonies were stained with crystal violet. [file 13046_2023_2776_MOESM5_ESM.pdf]

**A**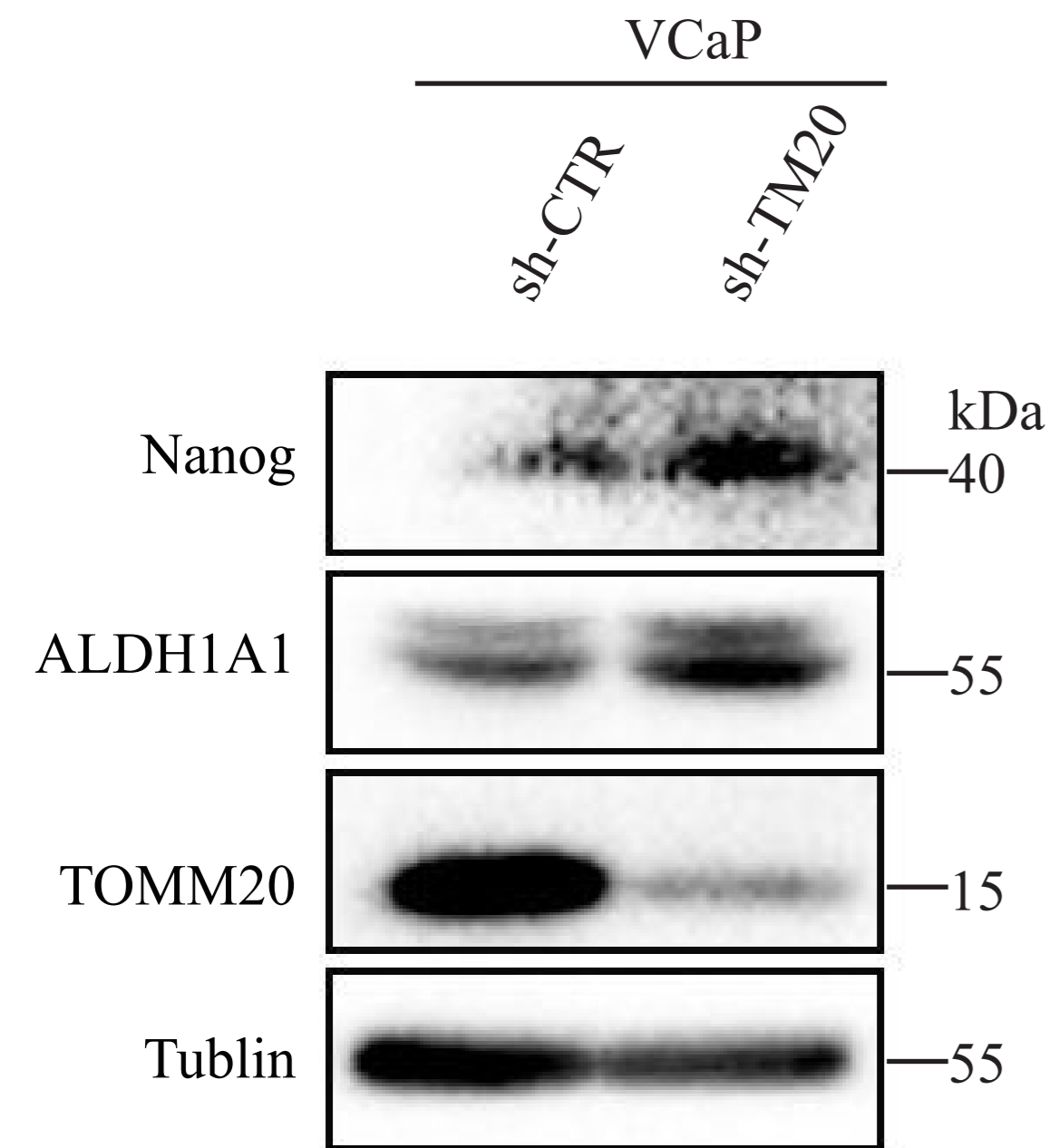**B**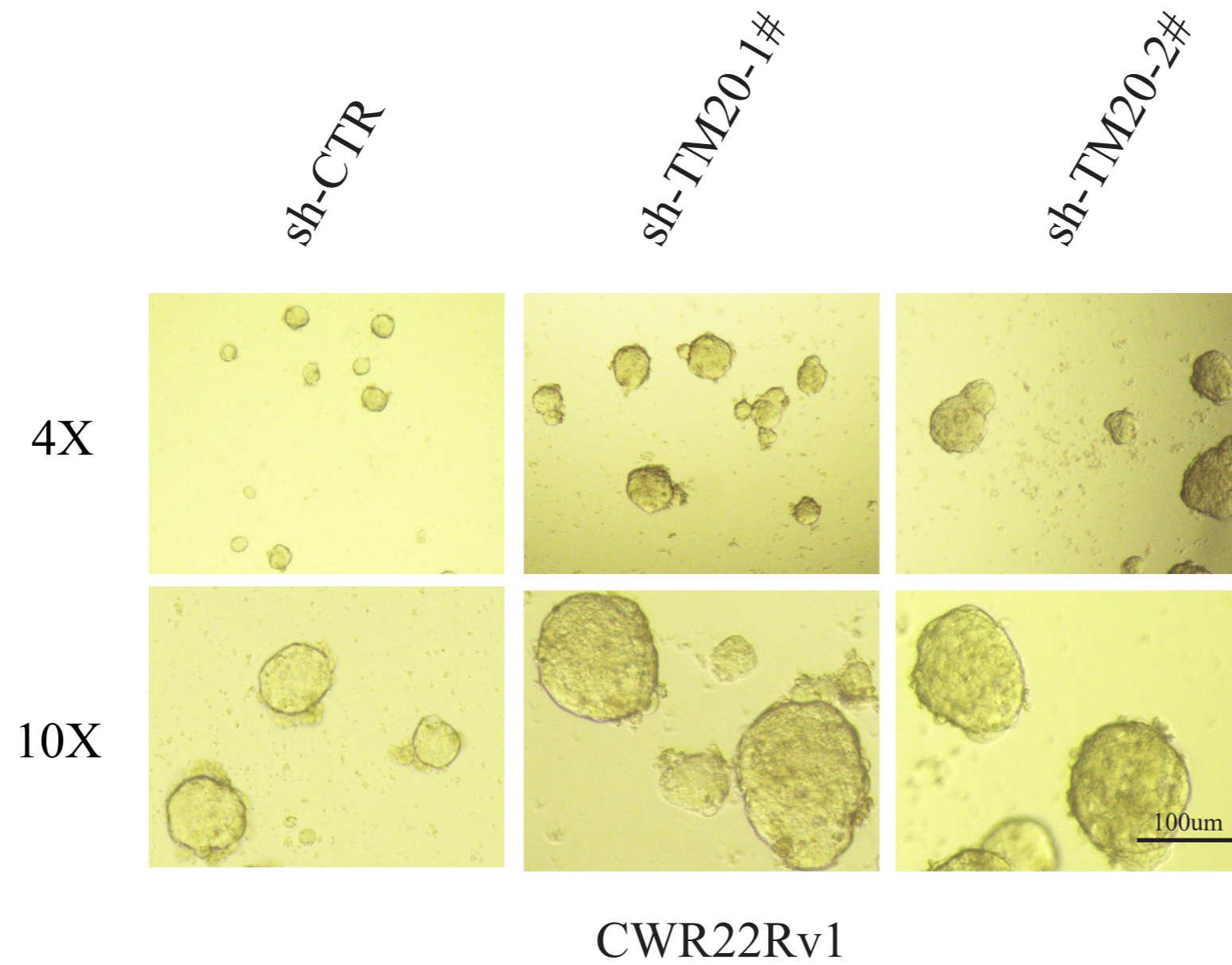**C**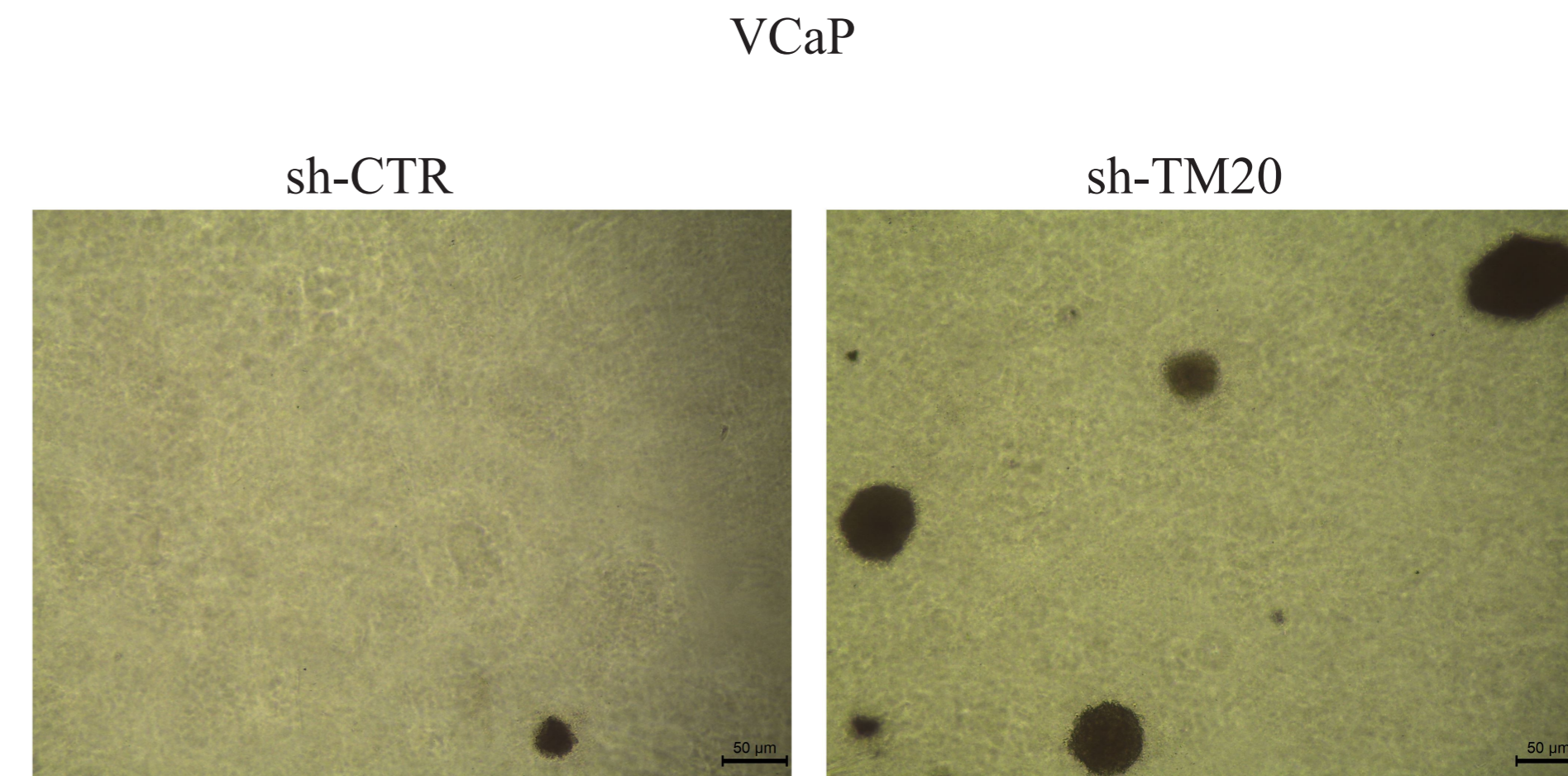

Supplement: Supplementary file 6 — Additional file 6: Supplementary Fig. 6. A VCaP cells stably expressing TOMM20 shRNA (shTOMM20) were established, and the protein levels of markers of cancer stem-like cells were assessed by western blotting. B. TOMM20 stably-depleted or parental CWR22Rv1 cells were diluted for cell spheroid assays. C. TOMM20 stably-depleted or parental VCaP cells were cultured in soft agar for two weeks, and cell morphology was observed by microscopy. [file 13046_2023_2776_MOESM6_ESM.pdf]

A

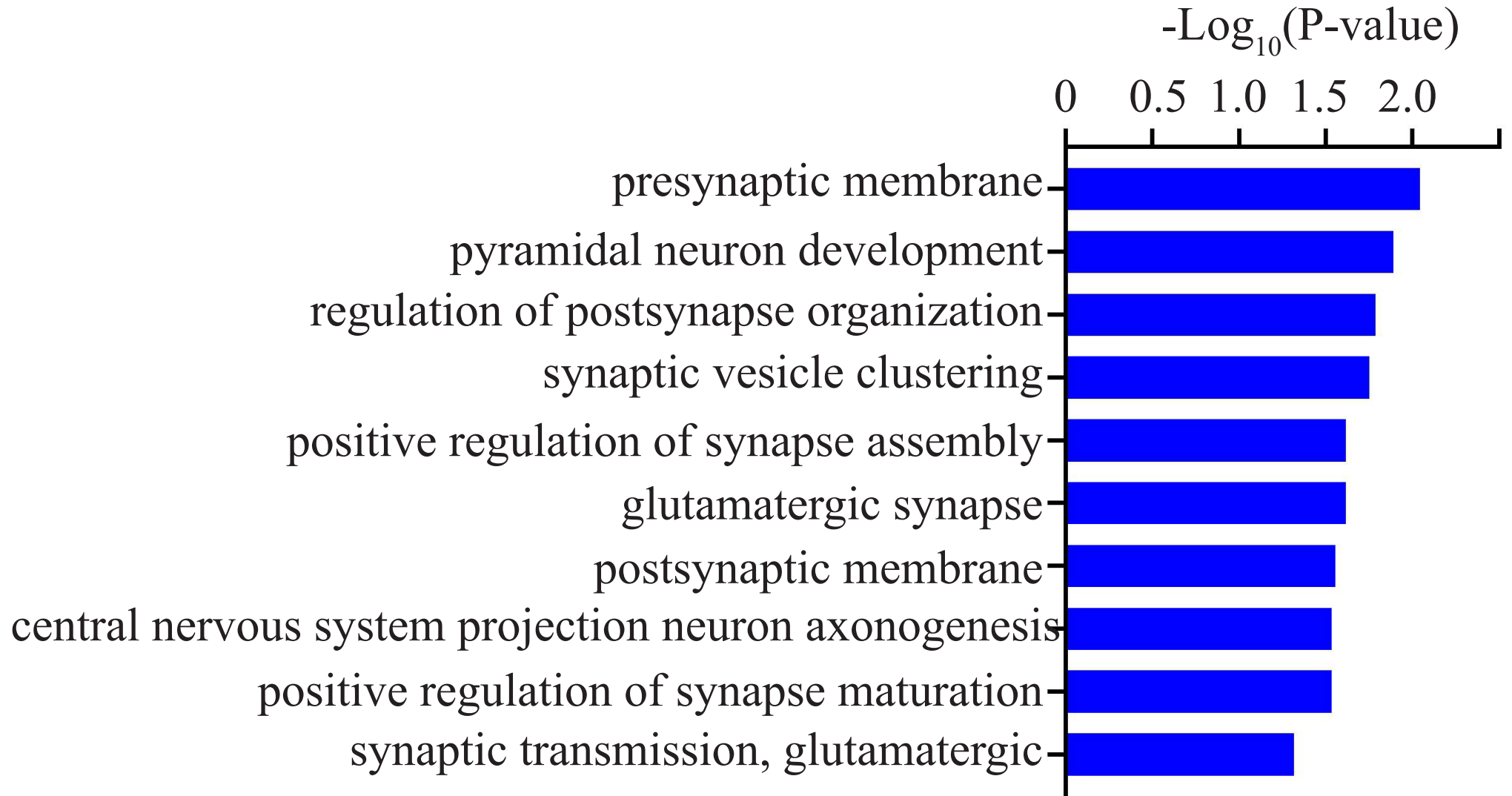

Supplement: Supplementary file 7 — Additional file 7: Supplementary Fig. 7. A GO analysis of RNA-seq data revealed that the neuronal-associated genes were up-regulated in TOMM20-depleted(sh-TM20) cells. [file 13046_2023_2776_MOESM7_ESM.pdf]
