# Supplementary material for: AR antagonists develop drug resistance through TOMM20 autophagic degradation-promoted transformation to neuroendocrine prostate cancer
Source: J Exp Clin Cancer Res. 2023 Aug 10;42:204. doi: 10.1186/s13046-023-02776-0 (PMC10413764; doi:10.1186/s13046-023-02776-0)
Supplement: Supplementary file 8 — Additional file 8: Supplementary Table. 1. Sequences of siRNA and RT-PCR primers used in this study. [file 13046_2023_2776_MOESM8_ESM.doc]

Sequences of siRNA used in this study

Negative control(sense:5′-UUCUCCGAACGUGUCACGUTT-3′)

si-AR-#1 (sense:5′-GACGCUUCUACCAGCUCACTT-3′),

si-AR-#2(sense:5′-CCAAUGUCAACUCCAGGAUTT-3′),

si-TOMM20-1#(5′-GGUCUUACAGCAAACUCUU-3′),

si-TOMM20-2#(5′-GCTGTTCAGAAGTTCTTCCTT-3′),

si-ATG5-#1(sense:5′-UGAACAGAAUCAUCCUUAATT-3′),

si-ATG5-#2(sense:5′-GATTCATGGAATTGAGCCAAT-3′).

RT-primers used in this study

| TOMM20-F-RT | CGACCGCAAAAGACGAAGTGAC |
| --- | --- |
| TOMM20-R-RT | GCTTCAGCATCTTTAAGGTCAGG |
| AR-F-RT | ATGGTGAGCAGAGTGCCCTATC |
| AR-R-RT | ATGGTCCCTGGCAGTCTCCAAA |
| KLK3-F-RT | CGCAAGTTCACCCTCAGAAGGT |
| KLK3-R-RT | GACGTGATACCTTGAAGCACACC |
| NCAM1-F-RT | CATCACCTGGAGGACTTCTACC |
| NCAM1-R-RT | CAGTGTACTGGATGCTCTTCAGG |
| CHGA-F-RT | GGTTCTTGAGAACCAGAGCAGC |
| CHGA-R-RT | GCTTCACCACTTTTCTCTGCCTC |
| NSE-F-RT | CTGTATCGCCACATTGCTCAGC |
| NSE-R-RT | AGCTTGTTGCCAGCATGAGAGC |
| SYP-F-RT | TCGGCTTTGTGAAGGTGCTGCA |
| SYP-R-RT | TCACTCTCGGTCTTGTTGGCAC |
| SYN-F-RT | CGATGCCAAATATGACGTGCGTG |
| SYN-R-RT | AGCATCGCAGAGCCAGTATTGG |
| ALDH1A1-F-RT | CGGGAAAAGCAATCTGAAGAGGG |
| ALDH1A1-R-RT | GATGCGGCTATACAACACTGGC |
| NANOG-F-RT | CTCCAACATCCTGAACCTCAGC |
| NANOG-R-RT | CGTCACACCATTGCTATTCTTCG |
| SOX2-F-RT | GCTACAGCATGATGCAGGACCA |
| SOX2-R-RT | TCTGCGAGCTGGTCATGGAGTT |
| OCT4-F-RT | CCTGAAGCAGAAGAGGATCACC |
| OCT4-R-RT | AAAGCGGCAGATGGTCGTTTGG |
| GAPDH-F-RT | GTCTCCTCTGACTTCAACAGCG |
| GAPDH-R-RT | ACCACCCTGTTGCTGTAGCCAA |
